# Ultrastructural, Energy-Dispersive X-ray Spectroscopy, Chemical Study and LC-DAD-QToF Chemical Characterization of *Cetraria islandica* (L.) Ach

**DOI:** 10.3390/molecules28114493

**Published:** 2023-06-01

**Authors:** Nurlen Manassov, Mamdouh Nabil Samy, Ubaidilla Datkhayev, Bharathi Avula, Sebastian John Adams, Kumar Katragunta, Vijayasankar Raman, Ikhlas A. Khan, Samir A. Ross

**Affiliations:** 1 National Center for Natural Products Research, School of Pharmacy, The University of Mississippi, University, MS 38677, USA; nura134@mail.ru (N.M.); bavula@olemiss.edu (B.A.); jasabest@olemiss.edu (S.J.A.); kkatragu@olemiss.edu (K.K.); vraman@olemiss.edu (V.R.); khan@olemiss.edu (I.A.K.); 2 S.D. Asfendiyarov Kazakh National Medical University, School of Pharmacy, Almaty 050012, Kazakhstan; u.datkhayev@kaznmu.kz; 3 Department of Pharmacognosy, Faculty of Pharmacy, Minia University, Minia 61519, Egypt; mamdouh.eskandr@mu.edu.eg; 4 Division of Pharmacognosy, Department of BioMolecular Sciences, School of Pharmacy, The University of Mississippi, University, MS 38677, USA

**Keywords:** *Cetraria islandica*, Iceland moss, LC-QToF-MS analysis, fragmentation study, secondary metabolites identification, anatomy, elemental analysis, fluorescence, microscopy, SEM

## Abstract

The lichen *Cetraria islandica* (L.) Ach. has been used in traditional and modern medicines for its many biological properties such as immunological, immunomodulating, antioxidant, antimicrobial, and anti-inflammatory activities. This species is gaining popularity in the market, with interest from many industries for selling as medicines, dietary supplements, and daily herbal drinks. This study profiled the morpho-anatomical features by light, fluorescence, and scanning electron microscopy; conducted an elemental analysis using energy-dispersive X-ray spectroscopy; and phytochemical analysis was performed using high-resolution mass spectrometry combined with a liquid chromatography system (LC-DAD-QToF) of *C. islandica*. In total, 37 compounds were identified and characterized based on comparisons with the literature data, retention times, and their mass fragmentation mechanism/s. The identified compounds were classified under five different classes, i.e., depsidones, depsides, dibenzofurans, aliphatic acids, and others that contain simple organic acids in majority. Two major compounds (fumaroprotocetraric acid and cetraric acid) were identified in the aqueous ethanolic and ethanolic extracts of *C. islandica* lichen. This detailed morpho-anatomical, EDS spectroscopy, and the developed LC-DAD-QToF approach for *C. islandica* will be important for correct species identification and can serve as a useful tool for taxonomical validation and chemical characterization. Additionally, chemical study of the extract of *C. islandica* led to isolation and structural elucidation of nine compounds, namely cetraric acid (**1**), 9′-(*O*-methyl)protocetraric acid (**2**), usnic acid (**3**), ergosterol peroxide (**4**), oleic acid (**5**), palmitic acid (**6**), stearic acid (**7**), sucrose (**8**), and arabinitol (**9**).

## 1. Introduction

Lichens are complex organisms composed of fungus, the mycobiont, and one or more photosynthetic algae (green alga or cyanobacteria). The photobiont lives in a symbiotic relationship, occurring in temperate habitats to the most extreme environmental conditions in the arctic and subarctic areas of Europe and North America [1,2,3]. Recently, studies showed that the lichens live in more complex structures, such as tripartite lichens or those with more partners [4]. This organism was considered as lichenized fungi based on differentiated characteristics, such as superficial thallus, slow growth rate, and longevity of thalli and fruit bodies [5]. Over 20,000 species of lichens are known worldwide [6], and it is estimated that around 1000 of these known species are from central Asia, mainly including Kazakhstan [7]. The flora of Kazakhstan is known for its rich diversity of all kinds of plant species, including lichens. The sample *Cetraria islandica* (L.) Ach. (Family: Parmeliaceae) was collected from the Karaganda region, Karkaraly National Park, located at 1200 m above sea level. The climate of this area is moderately continental, the average annual air temperature is 2 °C, in winter −15 °C, and in summer 20 °C. The global distribution of this species is reported in the west and north of Iceland, northern Wales, England, Scotland, Southwestern Ireland, and other parts of the world [8]. This species is commonly known as true Iceland lichen or Iceland moss. *C. islandica* is used in treating and prophylaxis of chronic obstructive pulmonary disease (COPD), mainly tuberculosis [9]. *C. islandica* is also used in the food industry as an additive in sweet dishes [10], and traditionally it is used for preparing herbal tea and homemade decoctions for treating dry cough, a temporary loss of appetite, and oral mucosal problems [11]. The scientific recommendation made by the Committee on Herbal Medicinal Products (HMPC) states that *C. islandica* can be used in the preparations of demulcent (smoothing agents) for mouth and throat irritation and the associated dry cough, and to treat the temporary loss of appetite only in adults, adolescents, and children over 6 years and above [12]. The micromorphology and chemical variation in *C. islandica* and *C. ericetorum* were studied earlier [13]. The reported chemical compounds of *C. islandica* are chitin; lichenin; isolichenin; sucrose; mannitol galactomannan; lichen acids: fumarprotocetraric acid, cetraric acid, fumaric acid, citric acid, usnic acid, and others; and minerals: calcium, sodium, iron, bromine, etc. [9,14]. Earlier pharmacological studies using crude extracts of *C. islandica* have shown immunological, immunomodulating, and bacteriostatic properties. The lichenin-types β-(1-3) and -(1-4)-glucan (lichenan) are the only β glucans found in Parmeliaceae proven to have antiviral activity [14,15]. The lichen acids, such as fumaroprotocetraric acid (2.6–11.5%), protocetraric acid (0.2–0.3%), protolichesterinic acid (0.1–1.5%), and usnic acid (0.04% of trace amount) were reported in *C. islandica* [16,17,18,19,20]. Fumaroprotocetraric acid has shown potential neuroprotective properties [21] and usnic acid was proven to act as an antibiotic. The importance of this lichen is very high in traditional and modern therapeutic usage, especially as dietary supplements and pharmaceutical products. Thus, this manuscript emphasizes profiling the macro and microscopic characters for authentication purposes.

Apart from morphological studies, spot tests on the thallus/medulla and the application of chromatography followed by high-resolution mass-spectrometry-based methods or direct spectrometric methods are useful in the chemotaxonomic identification of the species along with dereplication strategies and secondary metabolite characterization [22,23,24]. Exploration of the secondary metabolites—including metabolites in minor composition—using high-resolution mass-spectrometry-based methods leads to novel molecule isolation [25]. This current method for *C. islandica* analysis employs different solvent extractions to understand the extraction efficiency of the solvents in relation to the number of secondary metabolites. In addition, this paper will aim to establish the fragmentation mechanisms of major secondary metabolites in order to understand their class of chemistry and fragmentation pathways for dereplication strategies. As a result, isolated major chemical constituents are presented in this study.

## 2. Results

### 2.1. Macro-Morphological Description

**Figure 1:**
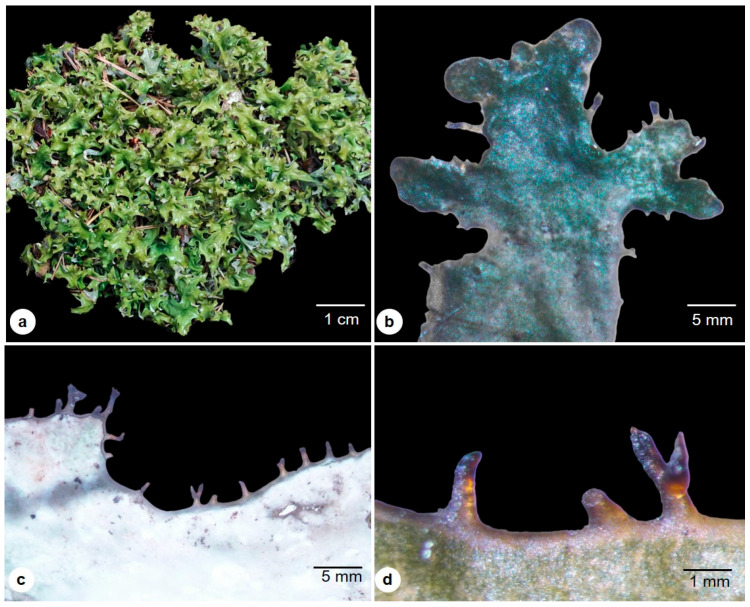
External morphology of *C. islandica*: (**a**) Habit; (**b**) Upper surface view; (**c**) Lower surface; (**d**) Brittle bands, Pycnidia.

This fruticose lichen is small to medium, with a heteromerous foliose thallus of 12–15 cm tall, upright, growing loose, irregular in structure, and attached to the substrate by the help of short filamentous rhizoids or its aggregate hyphae of the lower cortical layer of the thallus (Figure 1a). The thallus is glabrous, flat, or grooved, with brittle bands on blades of the thallus that are dichotomously branched, narrow at the base, curled ribbon-like at the top, with a width of 0.5 to 2 cm and less than 1 to 1.5 mm in diameter (Figure 1b,c). The fresh thallus is soft and leathery, pale white to silver on the lower surface and dark shiny green on the upper surface. In maturity, it turns dark greenish-brown on the upper surface and pale white to light gray on the lower surface (Figure 1c). The thallus is folded, wavy, and pitted in texture. Pycnidia are dark brown, ciliated at the margin, and uncommonly on the base of the branches (Figure 1d).

### 2.2. Chemical Spot-Test Analysis

**Figure 2:**
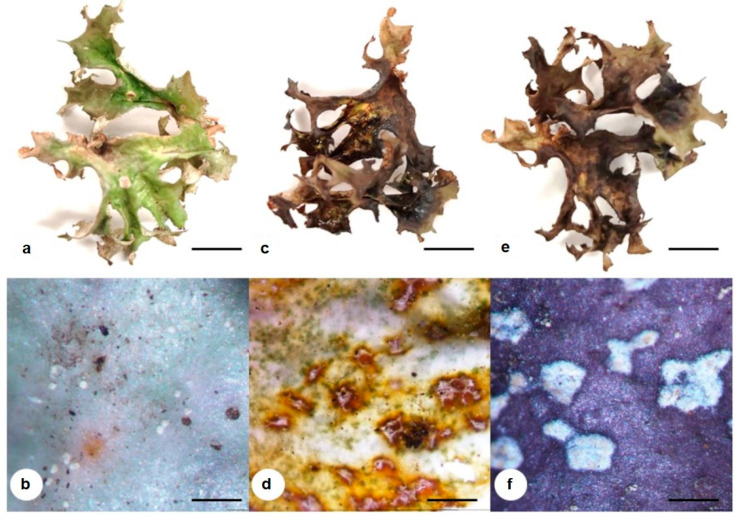
Chemical spot test of *C. islandica*, showing the color reaction after bleach (**a**,**b**), K (**c**,**d**), and I (**e**,**f**) tests. Scale bar: (**a**,**c**,**e**)—1 cm; (**b**,**d**,**f**)—0.5 mm.

The spot test suggests the presence of lichen acids, such as fumaroprotocetraric acid and protocetraric acid, by the K (potassium in the form of KOH) & I (Lugol’s solution) tests [16,18,20]. The K test gives the reddish-brown color changes in the upper and lower surface of the thallus, and the Iodine turns the thallus to bluish-black (Figure 2). These color changes are characteristic features of *C. islandica* due to its lichen acids.

### 2.3. Micro-Morphological Description

**Figure 3:**
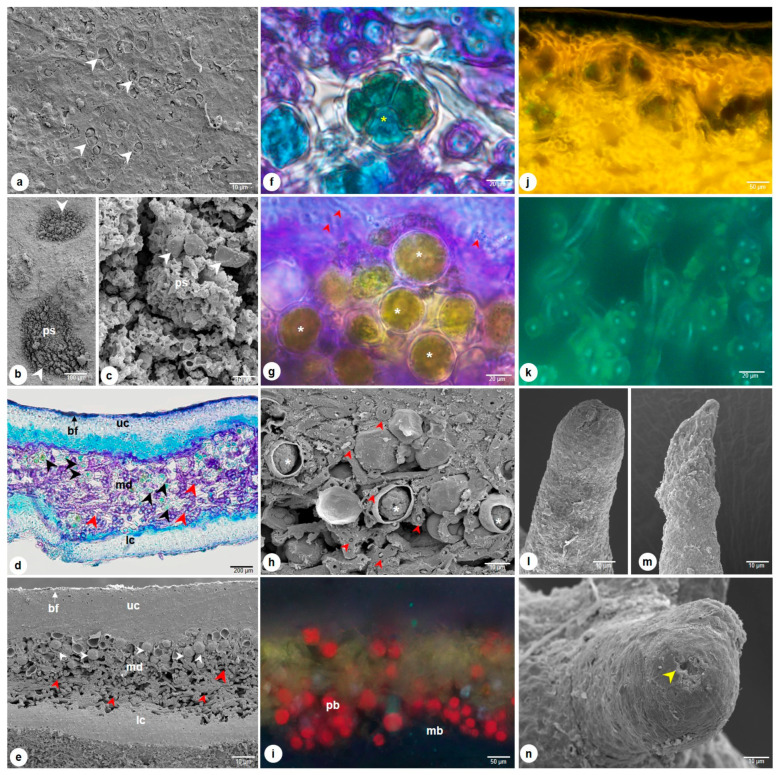
*C. islandica* thallus observed under light and scanning electron microscopy; (**a**) Upper surface, arrowhead indicating the opening of outer biofilm layer, cortical hyphae; (**b**,**c**) patches of pseudocyphella in lower surface (arrowhead); (**d**,**e**) Transverse section of thallus, showing the heteromerous structure of Mycobiont hyphae (red arrowhead) associated with algal cells (white & black arrowhead); (**f**) colony formation of daughter cells by asexual reproduction (yellow asterisk) after binary division; (**g**) The magnified view of phycobiont; (**h**) The mycelium (red arrowhead) and the chloroplast stroma (white asterisk) visible in open algal cells; (**i**,**j**) Autofluorescence of phycobiont (red) and mycobiont (yellow), highly differential with the specific emission of spectra of algal and fungal components; (**k**) Hyphal plasma autofluorescence in bluish-green and the cell wall in yellow; (**l**–**n**) Pycnidia; (**n**) Showing the ostioles (yellow arrowhead) at the apex. bf—biofilm; lc—lower cortex; mb—mycobiont; md—medulla; pb—phycobiont; ps—pseudocyphella; uc—upper cortex; Scale bars: 10 μm—(**a**,**c**,**e**,**l**–**n**); 20 μm—(**f**,**g**); 50 μm—(**i**,**j**); 100 μm—(**b**); 200 μm—(**d**).

The thallus is heteromeric, dorsoventral, and composed of dead, collapsed, gelatinized hyphae on both surfaces and the middle photobiont layer. The cortical upper region shows the surface opening, where the cortical hyphae emerge and help in gas exchanges (Figure 3a). The lower surface is uneven and grooved in many places, phycobiont and mycobiont hyphae are visible, and they are gonidial and cortical as well (Figure 3b). At the lower thallus, hyphae extend through the hydrophilic cortex layer and form a patch of pseudocyphella, showing many respiratory macules for air-breathing (Figure 3c); the ascomata are absent. The margin of the thallus with regular lobes of teeth-like structures, called pycnidia, is an asexual fruiting body. The transverse section is composed of an outer epicortex, 0.5–1.5 μm thick, and covered with biofilm (generally bacterial) (Figure 3d). The upper cortex, 30.05–50.6 μm wide, is formed into tight collectively packed cortical cells with fungal hyphae (Figure 3d,e). The medullary region measures 70–100 μm wide and consists of loosely interwoven mycobiont hyphae that run horizontally and provide large airspaces for the phycobiont. The algal cell, 3.25–12.5 μm in diameter, forms a bi-stratified structure. These phycobiont cells are located immediately below the upper cortex region of the medulla and are absent or lack abundance in the lower medulla region (Figure 3e). The binary division of reproduction is observed in the algal cell in the phycobiont region (Figure 3f). The internal chloroplast stroma is visible in the algal cell (Figure 3g,h), producing energy for its fungus counterpart. Mycobiont hyphae are 1.5–2 μm in width and are thick unicellular, forming a network of hydrated hyphae providing the water and growth environment to the algal cells. The unstained traverse section of hyphae shows the autofluorescence of phycobiont under the excitation band range of 340 to 390 nm, indicating the presence of chlorophyll pigments in the cells; and mycobiont visible under the excitation band range of 400 to 450 nm, due to the fact of the chitinous cell wall (Figure 3i,j) and the cross end of the hyphae visible with the outer cell wall (yellow) and inner plasma (in bluish green) under fluorescence (Figure 3k). The Pycnidia projected on the surface of lobes and the ostiole openings are visible under SEM observations (Figure 3l–n). The lower cortex region is about 25.05–33.05 μm in width, composed of tightly packed fungal hyphae.

### 2.4. EDS Mapping of Nano-Elemental Particles

**Figure 4:**
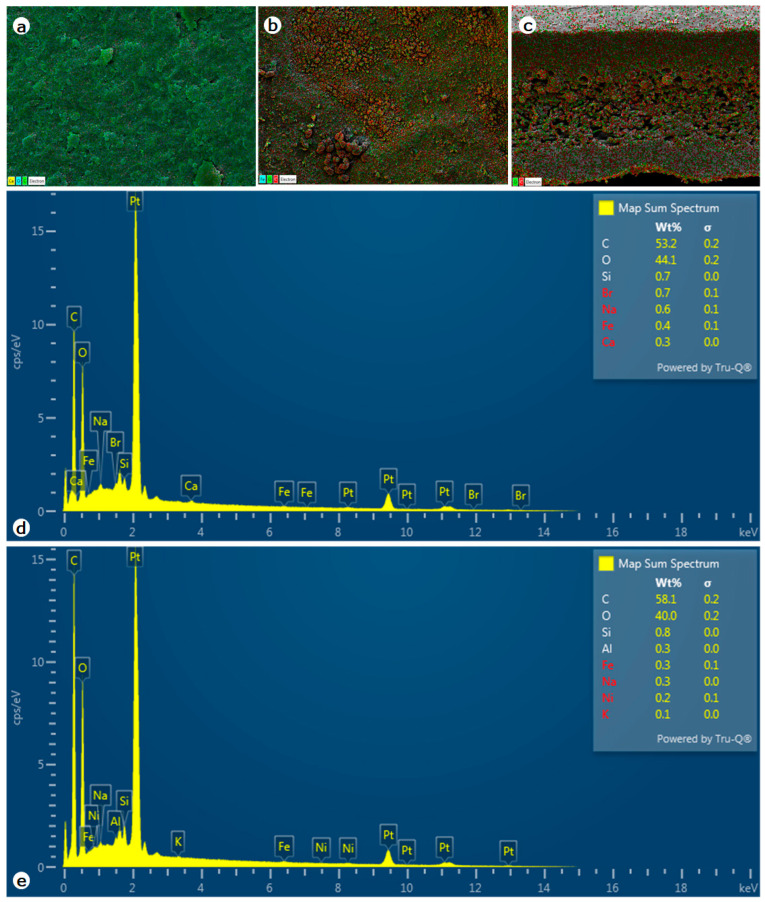
The EDS spectrum and elemental mapping of upper surface (**a**,**d**), lower surface (**b**,**e**), and transverse section of the *C. islandica* thallus (**c**).

Elemental mapping on the surface of *C. islandica* with the SEM micrograph shows the presence of some common elemental composition of calcium (Ca), and trace nano-sized crystalline silica (Si), bromine (Br), sodium (Na), iron (Fe) on the upper surface of the thallus (Figure 4a,d). These are in some homogeneous shapes with nanoparticle sizes. The lower surface showed the presence of silica (Si), sodium (Na), iron (Fe), nickel (Ni), potassium (K), and also calcium (Ca) (Figure 4b,c,e). In many lichens, it forms the calcium crystalline on the surface of the hyphal cell wall; this is not observed in this species. Secondly, sodium is present on the upper surface in trace amounts. Iron was detected on both surfaces of the thallus (Figure 4). *C. islandica* collected from Finland by Airaksinen et al. [17] noted minerals such as lead, cadmium, mercury, arsenic, calcium, magnesium, and iron in earlier studies. Most lichens have high metal accumulation due to their habitat; for example, the lichen collected from Iceland shows the maximum concentration of Co and P and the minimum of As, Cd, and Pb compared to other samples studied by Giordani et al. [26].

### 2.5. Identification of the Isolated Compounds from C. islandica

Chemical investigation of the extract of *C. islandica* led to the isolation and structural elucidation of nine compounds (Figure 5). The isolated compounds were identified as cetraric acid (**1**) [27], 9′-(*O*-methyl) protocetraric acid (**2**) [28], usnic acid (**3**) [29], ergosterol peroxide (**4**) [30], oleic acid (**5**), palmitic acid (**6**), stearic acid (**7**) [31], sucrose (**8**) [32], and D-arabinitol (**9**) [33]. The structures of the isolated compounds were determined using various spectroscopic analyses such as 1D NMR (^1^H NMR, ^13^C NMR, and DEPT), 2D NMR (^1^H–^1^H COSY, HSQC and HMBC), HR-ESI-MS, and GC-MS (Supplementary Material Figures S1–S28), and by comparing their chemical shifts with those reported in the literature.

### 2.6. Identification and Characterization of Major Lichen Acids and Other Compounds

**Figure 6:**
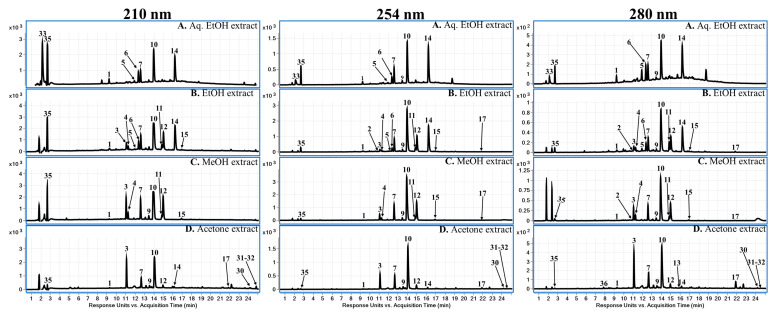
LC–DAD chromatograms for *Cetraria islandica* lichen extracts: (**A**) Aqueous EtOH; (**B**) EtOH; (**C**) MeOH; (**D**) Acetone at different wavelengths.

**Figure 7:**
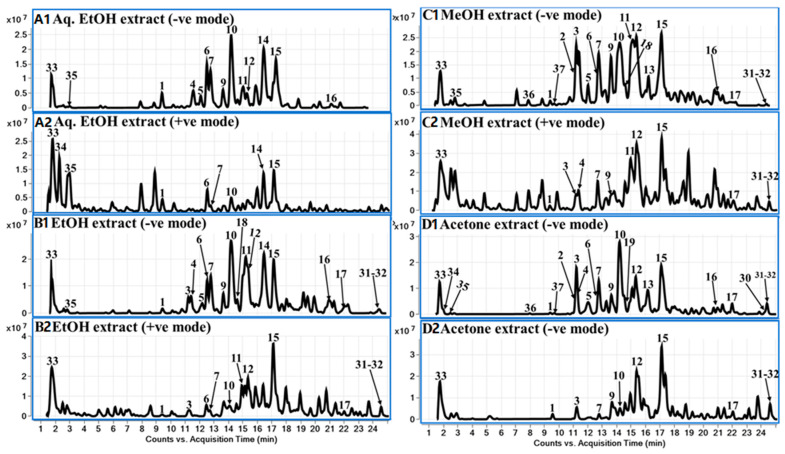
TCC (LC-QToF-ESI-MS) chromatograms for *Cetraria islandica* lichen extracts ((**A**) Aqueous EtOH; (**B**) EtOH; (**C**) MeOH; (**D**) Acetone).

Chemical constituents of *C. islandica* were analyzed by reverse phase LC using a gradient elution of the mobile phase. Further identification of chromatographic peaks using electrospray ionization–high-resolution mass-spectrometry (ESI–QToF), was carried out for identification and characterization of chemical constituents including depsidones (**1**–**15**), depsides (**16**), dibenzofuran (**17**), aliphatic acids or lipids (**18**–**32**), and other acids, etc., (**33**–**37**). Data was obtained using both positive and negative ESI modes and suggested that the negative mode performed better than the positive mode. The identified compounds are summarized in Table 1, including the retention time, molecular formulas, *m/z* values in both positive and negative modes, and major HRMS mass fragment ions. A total of 61 metabolites were detected, and among them, 37 compounds were tentatively characterized based on accurate mass spectrum and fragment ions, while 24 peaks remained unidentified (Supplementary Material Figure S29). DAD chromatograms of various extracts (Aq. EtOH, EtOH, MeOH, and acetone) of *C. islandica* are represented in Figure 6 at 210 nm, 254 nm, and 280 nm wavelengths. The representative LC-MS total current chromatograms of *C. islandica* in the negative mode of ionization are presented in Figure 7. Further, we compared and analyzed the distribution of the secondary metabolites in different extracts of the *C. islandica* sample. Structures were tentatively assigned to secondary metabolites of *C. islandica*.

#### 2.6.1. Depsidones (Compounds **1**–**15**)

**Figure 8:**
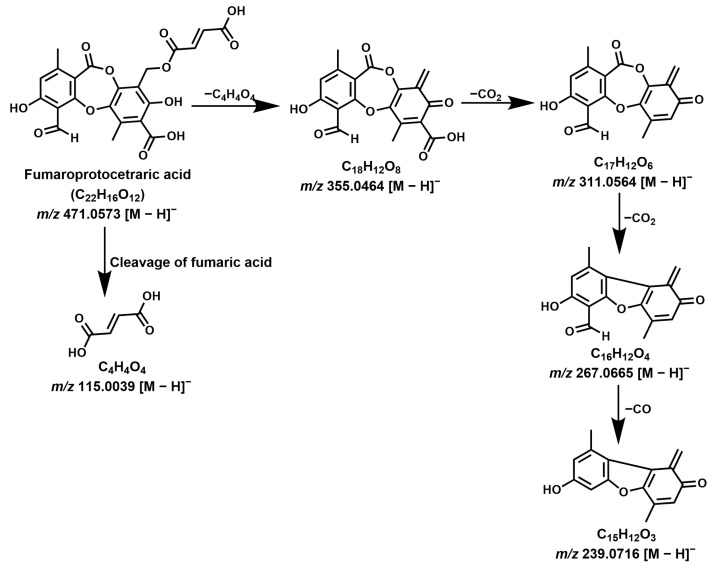
MS/MS fragmentation mechanism of fumaroprotocetraric acid (Compound **10**).

**Figure 9:**
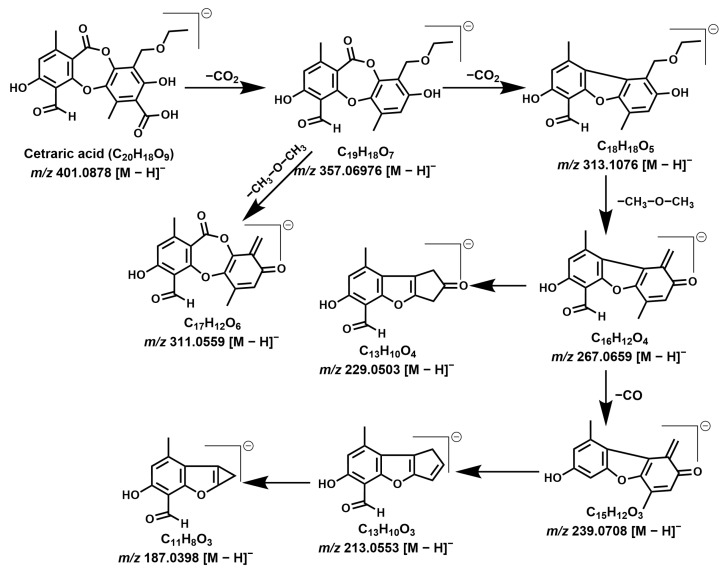
MS/MS fragmentation mechanism of cetraric acid (Compound **14**).

Twelve depsidones were tentatively identified corresponding to the peaks **1**–**15**. Gudjonsdottir and Ingólfsdóttir reported fumaroprotocetraric acid as the major compound, belonging to the depsidone class [20]. Protocetraric acid derivatives are the major compounds observed among various *C. islandica* extracts. Protocetraric acid (compound **7**, *m/z* 373.0560, C_18_H_13_O_9_), commonly found in the *Ramalina* genus, was detected in *C. islandica* samples [34]. Major product ion/s correspond to *m/z* 329.0666 [M-H-CO_2_]^−^, 311.0561 [M-H-CO_2_-H_2_O]^−^, 285.0765 [M-H-2CO_2_]^−^, and 255.0665 [M-H-2CO_2_-OCH_2_]^−^ indicate the sequential loss of -CO_2_, -H_2_O, and -OCH_2_ groups. This explains the presence of protocetraric acid with precursor ion 373.0570 [M − H]^−^ in the negative mode ionization. Because of its acidic nature, compounds favor the negative mode of ionization rather than the positive mode. Two major compounds, at 14.2 min and 16.5 min, were identified in this study assigned as fumaroprotocetraric acid (compound **10**) and cetraric acid (compound **14**), respectively. The detailed fragmentation mechanism (Figure 8 and Figure 9) is based on the observed product ions that are shown in Table 1. Based on this, above the fragmentation mechanism, the chemical structures of compounds **1**–**15** were tentatively assigned [22].

#### 2.6.2. Depside/s (Compound **16**)

Depsides are lichenic aromatic compounds formed by the esterification of two orcinol units [35]. Consequently, depside fragmentation usually gives rise to characteristic fragment ions due to the cleavage of the ester bond. Compound **16,** with a [M − H]^−^ pseudomolecular ion at *m/z* 387.1449, was identified as divaricatic acid, which showed diagnostic fragment ions at *m/z* 209.0822, 195.0662 [M-H-C_11_H_12_O_3_]^−^, 177.0556 [M-H-C_11_H_12_O_3_-H_2_O]^−^, and 151.0765 [M-H-C_11_H_12_O_3_-CO_2_]^−^. The fragment ions at *m/z* 209.0822 and 195.0662 are due to the cleavage of the ester bond.

#### 2.6.3. Dibenzofurans (Compound **17**)

Usnic acid (Compound **17**) is the only compound belonging to dibenzofurans in this study. The precursor ion at *m/z* 343.0823 [M − H]^−^ belongs to usnic acid, which was confirmed by the MS/MS fragments observed, as shown in Table 1. Characteristic fragments of usnic acid show *m/z* 328.0586 [M-H-CH_3_]^−^, *m/z* 259.0606 generated from a precursor ion through Retro Diels Alder (RDA) cleavage of C1–12 and C2–3 bonds, accompanied by the loss of characteristic fragments at *m/z* 84 Da, which subsequently leads to the loss of a -CO moiety produced fragment at *m/z* 231.0660 [23].

#### 2.6.4. Aliphatic Acids/Lipids (Compounds **18**–**32**)

Gudjonsdottir et al. and Xu et al. reported that aliphatic acids are another major class of compounds present in *C. islandica* [20,34]. The fragmentation pathway and profiles of product ions for compounds **18**–**32** were similar in each case. Compounds **18**–**29** are polyhydroxylated lipids, which are reported previously [23,36] from various lichens. Precursor ions—[M − H]^−^ with 403.3069 (C_22_H_44_O_6_), 417.3233 (C_23_H_46_O_6_), 431.3384 (C_24_H_48_O_6_), 459.3696 (C_26_H_52_O_6_), 251.1020 (C_16_H_28_O_2_), and 385.2595 (C_21_H_38_O_6_)—were identified as ventosic acid, tetrahydroxytrocosanoic acid, 6-ethyl-6-*n*-pentylpentadecan-4,5,7,8,15-pentol-15-acetate [37], tetrahydroxyhexasanoic acid, hexadecadienoic acid, and rangiformic acid, respectively, based on the exact mass and their fragment ions observed (Table 1). Since the compound structures were tentatively proposed as per Reddy et al., compounds **19**, **21**–**24,** and **26** were mentioned as unreported compounds based on their molecular formula and fragment ions observed [23]. Further, some of these aliphatic acids contain long carbon chains attached to a furan ring with electron-withdrawing groups (i.e., -COOH, -C=O) (compounds **30**–**32**) were identified as roccellaric acid, lichesterinic acid/protolichesterinic acid, respectively. Aliphatic compounds follow the sequential loss of CO_2_ and cleavage of carbon linkage.

#### 2.6.5. Others (Compounds **33**–**37**)

Apart from depsidones, depsides, dibenzofurans, and aliphatic acid, few other compounds were identified in the *C. islandica* extracts. Most of these compounds are citric acid, pyroglutamic acid, fumaric acid, and benzoic acid. Identified compounds along with their exact mass and fragment ions are provided in Table 1.

## 3. Discussion

The thalli consist of densely anastomosing hyphae in the upper and lower cortex and loosely formed hyphae around the phycobiont in the medullary region. The phycobiont in this species is the unicellular green algae *Trebouxia erici* [38], while the mycobiont fungus belongs to the genus *Aspicilia* [39]. The fungal partner is more responsible for the nutritional supply to the algal partner and maintains their hydration. Thus, this lichen behaves poikilohydric to survive unfavorable conditions [40,41,42]. Morphologically, the main characteristic to distinguish *C. islandica* from *closely related lichen* is the presence of distinct pseudocyphellae on the lower surface; this observation is similar to the earlier description by Honegger and Haisch [43], this characteristic is also used to distinguish *C. islandica var. islandica* and *C. islandica var. tenuifolia* by the region of presence [44]. Most lichens produce a wide range of polyphenols, which crystallize at hypha surfaces in the medullary layer or within the peripheral cortex, giving the thalli a characteristic coloration [45]; however, these features are not observed in this species. This lichen is attached to the substrate mainly by the clusters of agglutinated parallel hyphae at the basal region. The EDS spectra reveal that calcium and sodium are present on the upper surface alone. Iron elements are common on both surfaces. Due to their capability of accumulating inorganic minerals from the environment, lichens are widely used as indicators of air pollution or air quality. The analysis of heavy metals on the samples collected from different regions needs to be taken into consideration to avoid the risk factors in humans [46]. These elemental studies also significantly help to know the spatial relationship between the lichen and its habitat as well as its environmental condition.

LC-MS is a powerful tool for the qualitative analysis of secondary metabolites from lichens [47]. Structures of lichen secondary metabolites are characterized by the presence of carboxyl or phenyl groups, which could be ionized by ESI [22,23]. In this present study, 37 compounds were clearly tentatively identified and characterized from *C. islandica* by LC-QToF-MS/MS by accurate mass, molecular formula, and fragmentation pattern.

The most abundant secondary metabolites from the *Cetraria* species are polyketides and aliphatic acids. Dibenzofuran derivatives such as usnic acid, depsidones such as fumarprotocetraric and protocetraric acids, and fatty acids such as lichesterinic and protolichesterinic acids, are present in *Cetraria* species [48].

Protocetraric acid derivatives are the major compounds observed among various *C. islandica* extracts. Protocetraric acid (Compound **7**) was commonly found in the *Ramalina* genus [27] and *C. islandica* [48]. Two major compounds were identified in this study, assigned as fumaroprotocetraric acid (Compound **10**) and cetraric acid (Compound **14**), found in *C. islandica* [48]. On the other hand, usnic acid (Compound **17**) is the only compound belonging to dibenzofurans that was identified in this study and found in *C. islandica* [48,49].

## 4. Materials and Methods

### 4.1. General Experimental Procedures

^1^H and ^13^C NMR spectra were recorded on Bruker Avance 400 MHz instrument. HR-ESI-MS was taken on BrukerBioApex-FTMS with electron spray ionization. Solvents used in this work, e.g., *n*-hexane, dichloromethane (DCM), ethyl acetate (EtOAc), methanol (MeOH), and ethanol (EtOH), were purchased from Fisher Scientific, USA. Deuterated solvents including chloroform-d (CDCl_3_), methanol-d_4_ (CD_3_OD), and dimethyl sulfoxide-d_6_ (DMSO-d_6_)) were used for nuclear magnetic resonance (NMR) spectroscopic analyses purchased from Cambridge Isotope Laboratories, Inc., Tewksbury, MA, USA. Column chromatography (CC) was performed using silica gel 60 (Merck, Darmstadt, Germany; 70–230 mesh) and sephadex LH-20 (0.25–0.1 mm, Sorbent Technologies, Norcross, GA, USA). Thin-layer chromatography (TLC) analyses were carried out using pre-coated silica G plates w/UV_254_ (Sorbent Technologies, Norcross, GA, USA; 20 cm × 20 cm, 200 µm in thickness). Ultraviolet lamp (Spectroline ENF-240C, Specrtonics Corporation, New York, NY, USA) was used for visualization of spots on thin-layer chromatograms at 254 and/or 365 nm. Spots were visualized by spraying with 2% vanillin (Tokyo Chemical Industry Co. Ltd., Tokyo, Japan) in sulfuric acid-ethanol followed by heating at 110 °C.

### 4.2. Sample Collection

*Cetraria islandica* was collected in the region of the Republic of Kazakhstan, Karagandy Province, Karkaraly National Park, during the summer of 2021. The sample was assigned with NCNPR #24269 and deposited in the Botanical Repository of the National Center for Natural Product Research at the University of Mississippi. This specimen was found near the famous lake in this region—Shaitankol—growing on the fallen pine needles litter in the pine forest. *C. islandica* was authenticated by botanist Dr. Sebastian John Adams, National Center for Natural Products Research (NCNPR), University of Mississippi, USA, and also identified by Prof. Bruce McCune, Alumni Association Distinguished Professor, Department of Botany and Plant Pathology, Oregon State University, Corvallis, OR, USA.

### 4.3. Spot Test Procedure

The sample was subject to spot tests using sodium hypochlorite (bleach), potassium hydroxide (K-Test), and iodine (I-Test) on the surface of the *C. islandica*, and the color changes were recorded. These general procedures were followed as Microchemical Methods for identifying lichens by Orange [50].

### 4.4. Preparation of Samples for Light Microscopy

Microtome and hand-sections of fresh and dried samples were used for studies. Transverse sections of thalli by microtome were processed and stained with toluidine blue for histology observations and the hand sections for autofluorescence observations [51]. The micrographs were prepared using an Olympus BX53 fluorescence microscope equipped with DP74 camera systems and CellSens standard version imaging software (Olympus Corp., Tokyo, Japan). External morphology was photographed using a Nikon SMZ-U equipped with Nikon DSFiv Camera systems and Nikon Element BR Imaging Software (Nikon Inc., Tokyo, Japan).

### 4.5. Preparation of Samples for Scanning Electron Microscopy (SEM) & Energy-Dispersive X-ray Spectroscopy (EDS)

The samples were rehydrated with water in an oven at 60 °C, fixed in 2.5% glutaraldehyde for two days, washed in sodium cacodylate buffer, and then passed through 30%, 50%, 70%, 90%, and 100% ethanol solutions. The samples were then dried in a Leica CPD300 critical point dryer (Leica Microsystems, Wetzlar, Germany) supplied with liquid CO_2_. The dried samples were mounted on aluminum stubs with double-sided adhesive carbon tapes and coated with platinum using a Desk V TSC sputter coater (Denton Vacuum, Moorestown, NJ, USA) supplied with argon gas. The samples were imaged using a JSM-7200F field-emission SEM (JEOL Ltd., Tokyo, Japan). Mineral elements were mapped and analyzed using an EDS detector (Oxford Instruments, Oxford, UK) attached to the SEM.

### 4.6. Extraction and Isolation

The dried lichen material (570 g) was extracted by maceration with 95% ethanol three times at room temperature. The combined extracts were concentrated under reduced pressure to yield a residue (27.41 g). The ethanolic extract (18.73 g) was fractionated by using silica gel VLC technique, in which it was eluted initially with DCM, DCM-MeOH (5% and 10%), and then with EtOAc-DCM-MeOH-H_2_O (15:8:4:1, 10:6:4:1, 6:4:4:1) and finally washed with MeOH affording 29 fractions. Fraction 7 (975.6 mg) was subjected to Sephadex LH-20 CC using MeOH, yielding 8 subfractions. The second subfraction (860.8 mg) was purified through silica gel CC using hexane-EtOAc (20, 25, 30, and 35%), then DCM and DCM-MeOH (5%), giving a mixture of compounds **5**, **6,** and **7** (53.8 mg), and compound **4** (82.3 mg). Fraction 14 (1.42 g) was rechromatographed over silica gel CC using EtOAc-DCM-MeOH-H_2_O (15:8:4:1 and 10:6:4:1) and finally washed with MeOH, producing compound **1** (47.6 mg) and compound **3** (22.5 mg). Fraction 22 (2.38 g) was subjected to silica gel CC using EtOAc-DCM-MeOH-H_2_O (15:8:4:1, 10:6:4:1, and 6:4:4:1), then DCM-MeOH-H_2_O (65:35:10), and finally washed with MeOH, yielding compound **5** (18 mg) and compound **2** (34.8 mg). Compounds **8** and **9** were obtained from fraction 27 (4.90 g) and fraction 29 (1.58 g), respectively.

### 4.7. Liquid Chromatography-Diode Array Detector-Quadrupole Time-of-Flight Mass Spectrometry (LC-DAD-QToF)

#### 4.7.1. Chemical Used for LC-DAD-QToF Analysis

Acetonitrile, methanol, formic acid used are of HPLC-certified grade and water was purified using a Milli-Q system (Millipore, Bedford, MA, USA).

#### 4.7.2. Sample Preparation for LC-DAD-QToF Analysis

One gram of shade-dried *C. islandica* lichen material was weighed and extracted with aqueous ethanol, ethanol, methanol, and acetone solvents individually using 3 mL respective solvent followed by centrifugation at 10,000 rpm for 15 min. The extraction procedure was repeated for total of three times to ensure the maximum amount to be extracted from lichen samples. Further final volume was adjusted to 10 mL using respective solvents used for extraction. In continuation, the solvent was evaporated under vacuum conditions. From this, 10 mg of dried extract was weighed and dissolved in methanol solvent followed by filtration using 0.20 µ PTFE syringe filters and placed into LC vials prior to analysis.

#### 4.7.3. Instrumentation Setup for Liquid Chromatography Diode Array Detector-Quadrupole Time-of-Flight Mass Spectrometry (LC-DAD-QToF)

The liquid chromatographic system was an Agilent Series 1290 and the separation was achieved on an Acquity UPLC^TM^ HSS C18 column (100 mm × 2.1 mm I.D., 1.8 µm). The mobile phase consisted of water with 0.1% formic acid (A) and acetonitrile with 0.1% formic acid (B) at a flow rate of 0.2 mL/min. Analysis was performed using the following gradient elution: 95% A/5% B to 85%A/15%B in 3 min; in next 17 min to 85 % B; and finally in next 3 min to 100% B. Each run was followed by a 3 min wash with 100% B and an equilibration period of 5 min with 95% A/5% B. Two microliters of sample were injected. The column temperature was 40 °C.

The mass spectrometric analysis was performed with a QToF-MS-MS (Model #G6545B, Agilent Technologies, Santa Clara, CA, USA) equipped with an ESI source with Jet Stream technology using the following parameters: drying gas (N_2_) flow rate, 13 L/min; drying gas temperature, 300 °C; nebulizer pressure, 20 psig; sheath gas temperature, 300 °C; sheath gas flow, 12 L/min; capillary voltage, 4000 V; nozzle voltage, 0 V; skimmer, 65 V; Oct RF V, 750 V; and fragmentor voltage, 150 V. All the operations, acquisition, and analysis of data were controlled by Agilent MassHunter Acquisition Software Ver. A.10.1 and processed with MassHunter Qualitative Analysis software Ver. B.10.00. Each sample was analyzed in positive mode over the range of *m/z* = 100–1100 and extended dynamic range (flight time to *m/z* 1700 at 2 GHz acquisition rate). Accurate mass measurements were obtained by means of reference ion correction using reference masses at *m/z* 121.0509 (protonated purine) and 922.0098 [protonated hexakis (1H, 1H, 3H-tetrafluoropropoxy) phosphazine or HP-921] in positive ion mode. Samples were analyzed in all-ion MS–MS mode, where experiment 1 was carried out with collision energy of zero and experiment two with fixed collision energy of 45 eV. The compounds were confirmed in each spectrum.

## 5. Conclusions

The morpho-anatomical features were described and this compliance of key diagnostic features will help the correct identification of raw materials in commerce and also serve the taxonomical and quality control in preparing the monograph. Additionally, nine compounds were isolated from *C. islandica*, including two depsidones, one benzofuran, two fatty acids, one sterol, and two sugars. On the other hand, metabolic profiling of C. islandica using LC-QToF led to the chemical characterization and identification of 37 compounds, of which depsidones were the major secondary metabolites, followed by aliphatic acids or lipids.

## Figures and Tables

**Figure 5:**
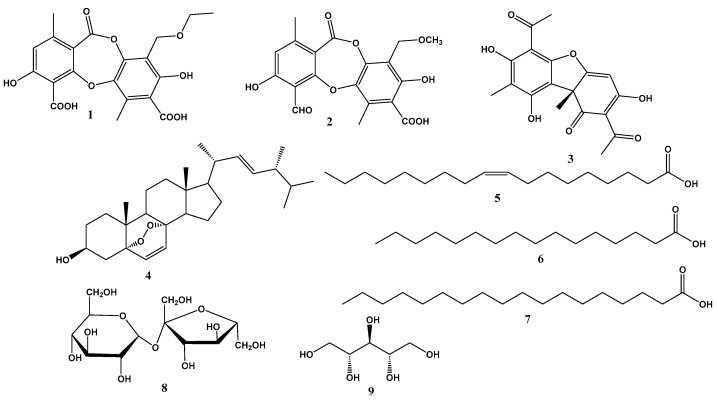
Structures of the isolated compounds from *C. islandica* (**1**–**9**).

**Table 1:** LC-QToF-MS data for compounds from different extracts of *Cetraria islandica* lichen.

#	RT (min)	Compound Name	Mol. Formula	Error (ppm)	[M − H]^−^	Fragment Ions (−ve Mode)	Extraction Solvent			
							Aq. EtOH	EtOH	MeOH	Acetone
**Depsidones**										
**1**	9.4	Dihydroprotocetraric acid	C_18_H_16_O_9_	0.53	375.0724	357.0622 [M-H-H_2_O]^−^; 313.0721 [M-H-H_2_O-CO_2_]^−^; 295.0612 [M-H-2H_2_O-CO_2_]^−^; 239.0716 [M-H-2H_2_O-CO_2_-2CO]^−^; 213.0557 [M-H-2H_2_O-CO_2_-2CO-CH_2_]^−^;	+	+	+	+
**2**	11.1	Dihydrosubpsoramic acid	C_17_H_14_O_8_	0.02	345.0616	327.0508 [M-H-H_2_O]^−^; 283.0612 [M-H-H_2_O-CO_2_]^−^, 239.0711 [M-H-H_2_O-2CO_2_]^−^;	+	+	+	+
**3**	11.2	Dihydrofumaroproto-cetraric acid	C_22_H_18_O_12_	0.63	473.0728	357.0620 [M-H-C_4_H_4_O_4_]^−^; 313.0722 [M-H-C_4_H_4_O_4_-CO_2_]^−^; 115.0040 [C_4_H_4_O_4_-H]^−^;	+	+	+	+
**4**	11.4	3,9-Dihydroxy-10-(hydroxymethyl)-4-(methoxymethyl)-1,7-dimethyl-6-oxobenzo[b][1,4]benzodioxepine-2-carboxylic acid	C_19_H_18_O_9_	0.00	389.0878	371.0774 [M-H-H_2_O]^−^; 357.0617 [M-H-H_2_O-CH_2_]^−^; 327.0873 [M-H-H_2_O-CO_2_]^−^; 313.0717 [M-H-H_2_O-CH_2_-CO_2_]^−^; 295.0615 [M-H-2H_2_O-CH_2_-CO_2_]^−^; 251.0714 [M-H-2H_2_O-CH_2_-2CO_2_]^−^;	ND	+	+	+
**5**	12.0	Methyl derivative of 3,9-Dihydroxy-10-(hydroxymethyl)-4-(methoxymethyl)-1,7-dimethyl-6-oxobenzo[b][1,4]benzodioxepine-2-carboxylic acid	C_20_H_20_O_9_	0.99	403.1039	385.0931 [M-H-H_2_O]^−^; 357.0618 [M-H-H_2_O-CH_2_-CH_3_]^−^; 313.0722 [M-H-H_2_O-CH_2_-CH_3_-CO_2_]^−^;	+	+	ND	ND
**6**	12.5						+	+	ND	ND
**7**	12.8	Protocetraric acid	C_18_H_14_O_9_	1.33	373.0570	355.0464 [M-H_2_O-H]^−^, 329.0666 [M-CO_2_-H]^−^; 311.0561 [M-H_2_O-CO_2_-H]^−^, 267.0664 [M-H_2_O-2CO_2_-H]^−^;	+	+	+	+
**8**	13.0	Physodalic acid	C_20_H_16_O_10_	0.24	415.0672	373.0567 [M-H-CH_2_-CO]^−^;	+	+	ND	ND
**9**	13.6	Succinprotocetraric acid	C_22_H_18_O_12_	0.42	473.0727	355.0461[M-H-C_4_H_4_O_4_]^−^; 311.0564 [M-H-C_4_H_4_O_4_-CO_2_]^−^; 239.0711 [M-H-C_4_H_4_O_4_-2CO_2_-CO]^−^; 117.0197 [C_4_H_6_O_4_-H]^−^;	+	+	+	+
**10**	14.2	Fumarprotocetraric acid	C_22_H_16_O_12_	0.85	471.0573	355.0464 [M-H-C_4_H_4_O_4_]^−^; 311.0565 [M-H-C_4_H_4_O_4_-CO_2_]^−^; 267.0665 [M-H-C_4_H_4_O_4_-2CO_2_]^−^; 239.0708 [M-H-C_4_H_4_O_4_-2CO_2_-CO]^−^; 115.0039 [C_4_H_4_O_4_-H]^−^;	+	+	+	+
**11**	15.1	Subpsoromic acid	C_17_H_12_O_8_	−1.16	343.0465	299.0566 [M-H-CO_2_]^−^; 255.0667 [M-H-2CO_2_]^−^; 229.0512 [M-H-2CO_2_-2CO]^−^; 213.0563 [M-H-C_4_H_2_O_5_]^−^; 201.0563 [M-H-C_5_H_2_O_5_]^−^;	+	+	+	+
**12**	15.3	Methylprotocetraric acid	C_19_H_16_O_9_	0.25	387.0723	343.0823 [M-H-CO_2_]^−^; 311.0562 [M-H-CO_2_-H_2_O-CH_2_]^−^; 267.0664 [M-H-2CO_2_-H_2_O-CH_2_]^−^; 255.0663 [M-H-2CO_2_-H_2_O-CH_2_-O]^−^; 239.0712 [M-H-2CO_2_-H_2_O-CH_2_-2O]^−^;	+	+	+	+
**13**	16.3	Vesuvianic acid	C_21_H_18_O_9_	−0.24	413.0877	355.0456 [M-H-C_3_H_6_O]^−^; 311.0560 [M-H-C_3_H_6_O-CO_2_]^−^;	ND	ND	ND	+
**14**	16.5	Cetraric acid	C_20_H_18_O_9_	−1.24	401.0883	357.0976 [M-H-CO_2_]^−^; 313.1076 [M-H-2CO_2_]^−^; 311.0561 [M-H-CO_2_-CH_3_-O-CH_3_]^−^; 267.0663 [M-H-2CO_2_-CH_3_-O-CH_3_]^−^; 239.0712 [M-H-2CO_2_-CH_3_-O-CH_3_-CO]^−^; 229.0508[M-H-2CO_2_-CH_3_-O-CH_3_-C_3_H_2_]^−^; 213.0558 [M-H-2CO_2_-CH_3_-O-CH_3_-CO-C_2_H_2_]^−^; 187.0400 [M-H-2CO_2_-CH_3_-O-CH_3_-CO-2C_2_H_2_]^−^;	+	+	+	+
**15**	17.1	Virensic acid	C_18_H_14_O_8_	1.40	357.0621	313.0718 [M-H-CO_2_]^−^; 269.0820 [M-H-2CO_2_]^−^;	+	+	+	+
**Depsides**										
**16**	20.7	Divaricatic acid	C_21_H_24_O_7_	0.00	387.1449	209.0822 [M-H-C_10_H_10_O_3_]^−^; 195.0662 [M-H-C_11_H_12_O_3_]^−^; 177.0556 [M-H-C_11_H_14_O_4_]^−^; 151.0765 [M-H-C_11_H_12_O_3_-CO_2_]^−^; 133.0657 [M-H-C_11_H_14_O_4_-CO_2_]^−^;	+	+	+	+
**Dibenzofuran/s**										
**17**	22.0	Usnic acid	C_18_H_16_O_7_	0.00	343.0823	328.0586 [M-H-CH_3_]^−^; 259.0608 [M-H-C_4_H_4_O_2_]^−^; 231.0660 [M-H-C_4_H_4_O_2_-CO]^−^;	+	+	+	+
**Aliphatic acids/Lipids**										
**18**	14.6	Ventosic acid	C_22_H_44_O_6_	0.99	403.3069	215.1288 [M-H-C_11_H_24_O_2_]^−^; 185.1183 [M-H-C_11_H_24_O_2_-OCH_2_]^−^; 169.1232 [M-H-C_11_H_24_O_2_-O-OCH_2_]^−^; 157.1233 [M-H-C_11_H_24_O_2_-C-O-OCH_2_]^−^;	+	+	+	+
**19**	14.89	Unreported compound	C_26_H_50_O_8_	1.43	489.3440	429.3224 [M-H-AcOH]^−^; 197.1548 [M-H-AcOH-C_12_H_24_O_4_]^−^; 167.1440 [M-H-AcOH-C_12_H_24_O_4_-CH_2_O]^−^; 157.1235 [M-H-AcOH-C_12_H_24_O_4_-3CH_2_]^−^; 127.1127 [M-H-AcOH-C_12_H_24_O_4_-3CH_2_-CH_2_O]^−^;	+	+	+	+
**20**	15.4	Tetrahydroxy tricosanoic acid	C_23_H_46_O_6_	2.63	417.3233	229.1448 [M-H-C_11_H_24_O_2_]^−^; 199.1341 [M-H-C_11_H_24_O_2_-CH_2_O]^−^; 183.1391 [M-H-C_11_H_24_O_2_-O-CH_2_O]^−^; 157.1235 [M-H-C_11_H_24_O_2_- CH_2_O-C_2_H_2_O]^−^; 127.1131 [M-H-C_11_H_24_O_2_- 2CH_2_O-C_2_H_2_O]^−^;	+	+	+	+
**21**	16.0	Unreported compound	C_27_H_52_O_8_	0.39	503.3591	443.3380 [M-H-AcOH]^−^; 293.1790 [M-H-AcOH-C_8_H_22_O_2_]^−^; 265.1478 [M-H-AcOH-C_8_H_22_O_2_-2CH_2_]^−^;	+	+	+	+
**22**	16.6						+	+	+	+
**23**	17.4						+	+	+	+
**24**	17.8						+	+	+	+
**25**	16.2	6-Ethyl-6-n-pentylpentadecan-4,5,7,8,15-pentol-15-acetate	C_24_H_48_O_6_	1.39	431.3384	243.1602 [M-H-C_11_H_24_O_2_]^−^; 213.1498 [M-H-C_11_H_24_O_2_-CH_2_O]^−^; 197.1545 [M-H-C_11_H_24_O_2_-O-CH_2_O]; 167.1440 [M-H-C_11_H_24_O_2_-O-2CH_2_O]; 157.1234 [M-H-C_11_H_24_O_2_-CH_2_O-C_3_H_4_O]^−^; 127.1130 [M-H-C_11_H_24_O_2_-2CH_2_O-C_3_H_4_O]^−^;	+	+	+	+
**26**	17.1	Unreported compound	C_28_H_54_O_8_	0.58	517.3749	457.3537 [M-H-AcOH]^−^; 241.1445 [M-H-AcOH-C_13_H_28_O_2_]^−^; 197.1528 [M-H-AcOH-C_13_H_28_O_2_-CO_2_]^−^; 185.1547 [M-H-AcOH-C_13_H_28_O_2_-C-CO_2_]^−^; 167.1441[M-H-AcOH-C_13_H_28_O_2_-C-CO_2_-H_2_O]^−^; 155.1442 [M-H-AcOH-C_13_H_28_O_2_-C-C-CO_2_-H_2_O]^−^;	+	+	+	+
**27**	18.4	Tetrahydroxy hexacosanoic acid	C_26_H_52_O_6_	1.09	459.3696	441.3579 [M-H-H_2_O]^−^; 351.2172 [M-H-H_2_O-C_6_H_18_]^−^;	+	+	+	+
**28**	22.5	Hexadecadienoic acid	C_16_H_28_O_2_	1.19	251.2020		ND	+	+	+
**29**	23.8	Rangiformic acid	C_21_H_38_O_6_	−0.26	385.2595	353.2330 [M-H-CH_3_OH]^−^, 309.2499 [M-H-CH_3_OH-CO_2_]^−^, 265.2536 [M-H-CH_3_OH-2CO_2_]^−^;	+	+	+	+
**30**	24.2	Roccellaric acid	C_19_H_34_O_4_	0.61	325.2386	281.2483 [M-H-CO_2_]^−^;	+	+	+	+
**31**	24.3	Lichesterinic acid/Protolichesterinic acid	C_19_H_32_O_4_	−0.31	323.2227	279.2326 [M-H-CO_2_]^−^;	+	+	+	+
**32**	24.5						+	+	+	+
**Others**										
**33**	2.0	Citric acid	C_6_H_8_O_7_	2.09	191.0201	111.0091 [M-CO_2_-2H_2_O]^−^;	+	+	+	+
**34**	2.2	Pyroglutamic acid	C_5_H_7_NO_3_	2.34	128.0356	-	ND	+	+	+
**35**	2.8	Fumaric acid	C_4_H_4_O_4_	0.00	115.0037	-	+	+	+	+
**36**	7.9	Benzoic acid	C_7_H_6_O_2_	0.00	121.0295	-	+	+	+	+
**37**	9.7	Diethylmethyl succinate	C_9_H_16_O_4_	0.53	187.0977	-	+	+	+	+

Note: + indicates presence of compound; ND—not detected. -CO_2_ = 43.9898 Da; -CH_3_OH = 32.0262 Da; -CH_3_ = 15.0235 Da; -CH_2_ = 14.0157 Da; -H_2_O = 18.0016 Da; -OCH_2_ = 30.0106 Da; -CO = 27.9949 Da; -C-CO = 39.9949 Da; -CH_3_-CH-CO = 56.0262 Da; -AcOH = 60.0211 Da.

## Data Availability

Not applicable.

## Supplementary Materials

The following supporting information can be downloaded at: https://www.mdpi.com/article/10.3390/molecules28114493/s1, Figures S1–S28: NMR, HR-ESI-MS, and EI-MS spectra of the isolated compounds **1**–**9**; Figure S29: HR-MS/MS spectra of the identified compounds **1**–**37**.

## Sample Availability

Samples of the compounds are not available from the authors.
