# Supplementary material for: Ultrastructural, Energy-Dispersive X-ray Spectroscopy, Chemical Study and LC-DAD-QToF Chemical Characterization of Cetraria islandica (L.) Ach
Source: Molecules. 2023 Jun 1;28(11):4493. doi: 10.3390/molecules28114493 (PMC10254383; doi:10.3390/molecules28114493)
Supplement: Supplementary file 1 [file molecules-28-04493-s001.zip › molecules-2408313-supplementary.pdf]

molecules

Supplementary Material

Nurlen Manassov [1,2], Mamdouh Nabil Samy [3], Ubaidilla Datkhayev [2], Bharathi Avula [1], Sebastian John Adams [1], Kumar Katragunta [1], Vijayasankar Raman [1], Ikhlas A. Khan [1,4] and Samir A. Ross [1,2,4,*]

1 National Center for Natural Products Research, School of Pharmacy, The University of Mississippi, University, MS 38677, USA; nura134@mail.ru (N.M.); bavula@olemiss.edu (B.A.); jasabest@olemiss.edu (S.J.A.); kkatragu@olemiss.edu (K.K.); vraman@olemiss.edu (V.R.); khan@olemiss.edu (I.A.K.)
2 S.D. Asfendiyarov Kazakh National Medical University, School of Pharmacy, Almaty 050012, Kazakhstan; u.datkhayev@kaznmu.kz
3 Department of Pharmacognosy, Faculty of Pharmacy, Minia University, Minia 61519, Egypt; mamdouh.eskandr@mu.edu.eg
4 Division of Pharmacognosy, Department of BioMolecular Sciences, School of Pharmacy, The University of Mississippi, University, MS 38677, USA
* Correspondence: sross@olemiss.edu

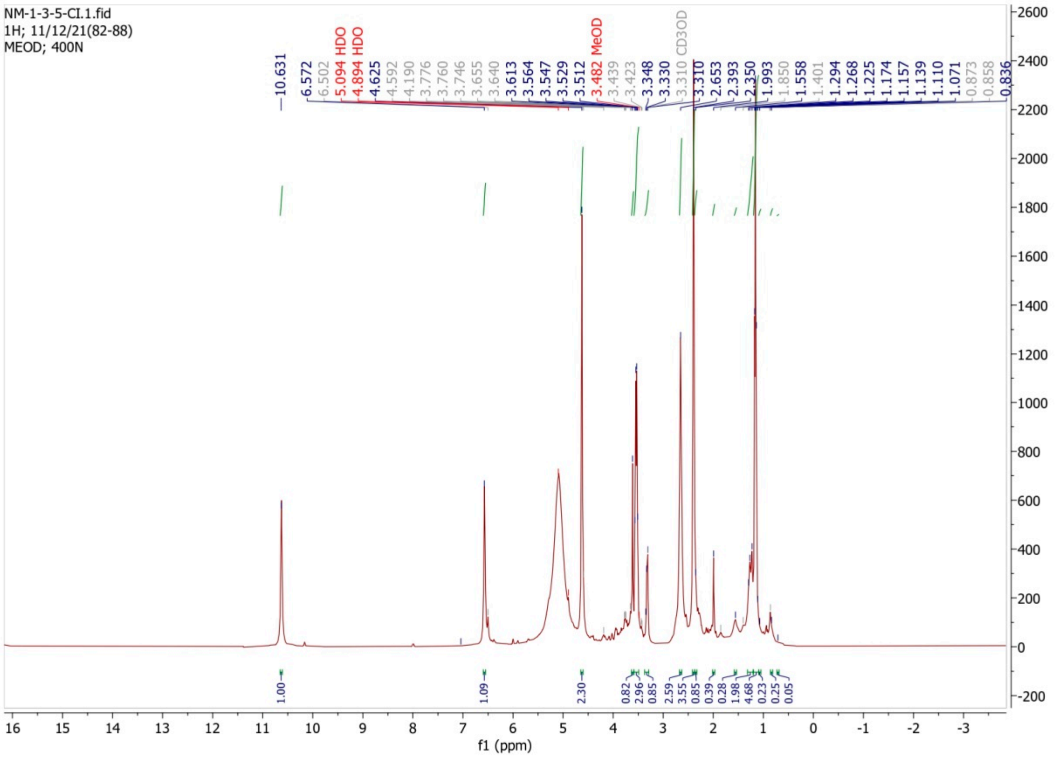

Figure S1. ^{1}H NMR spectrum of compound **1** (400 MHz, CD_3OD)

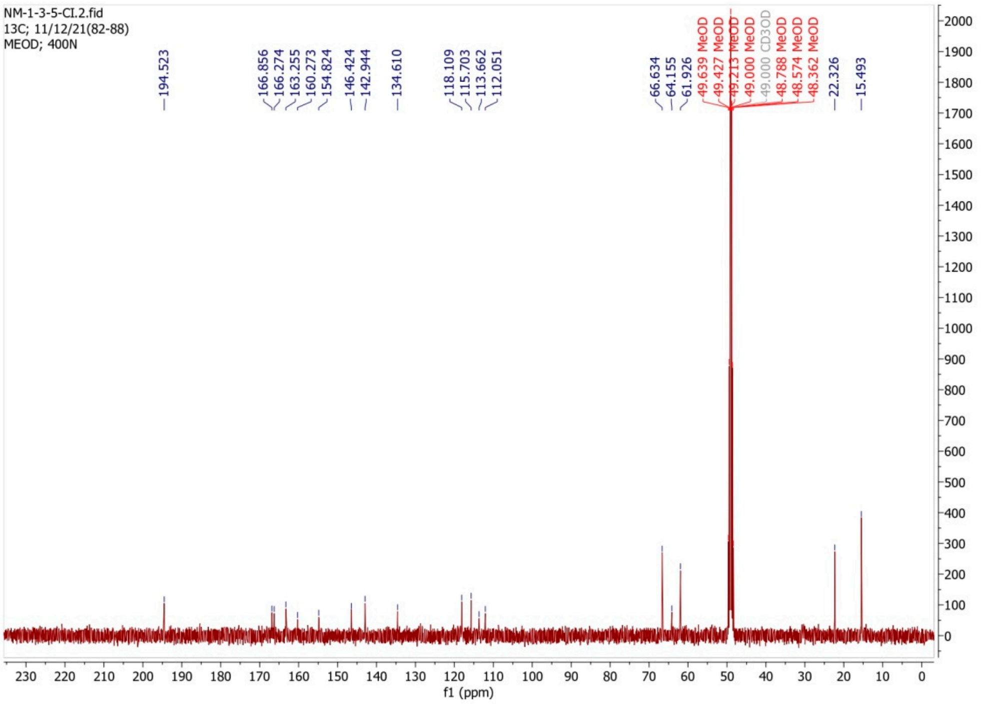

Figure S2. ^{13}C NMR spectrum of compound **1** (100 MHz, CD_3OD)

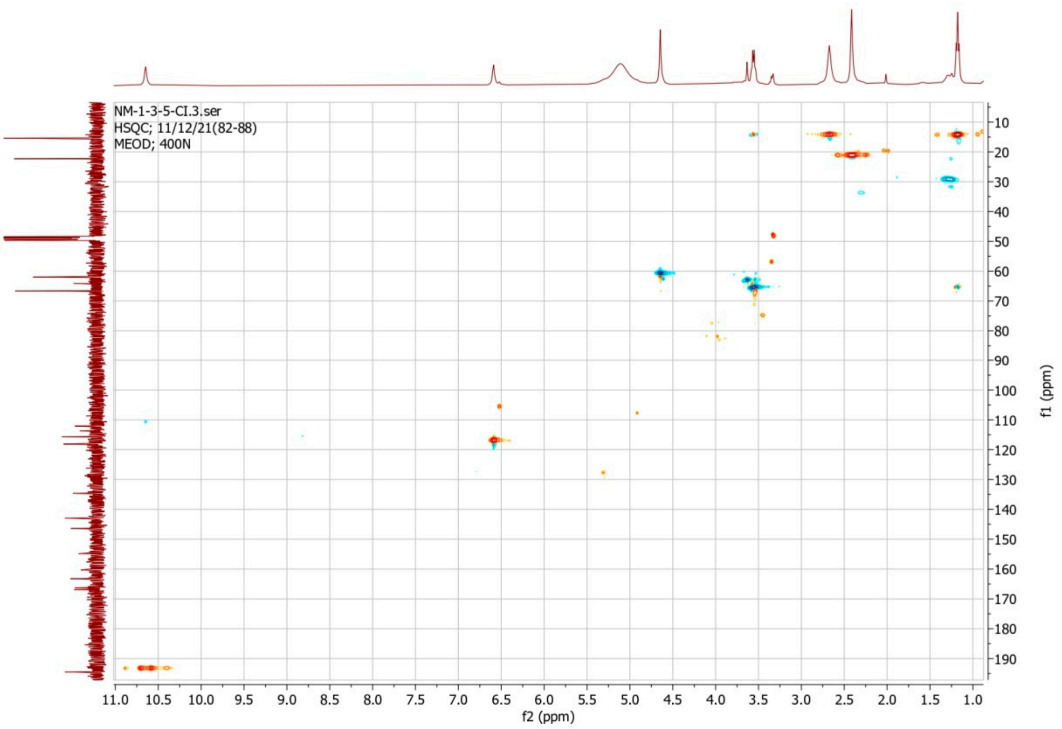

Figure S3. HSQC spectrum of compound **1**

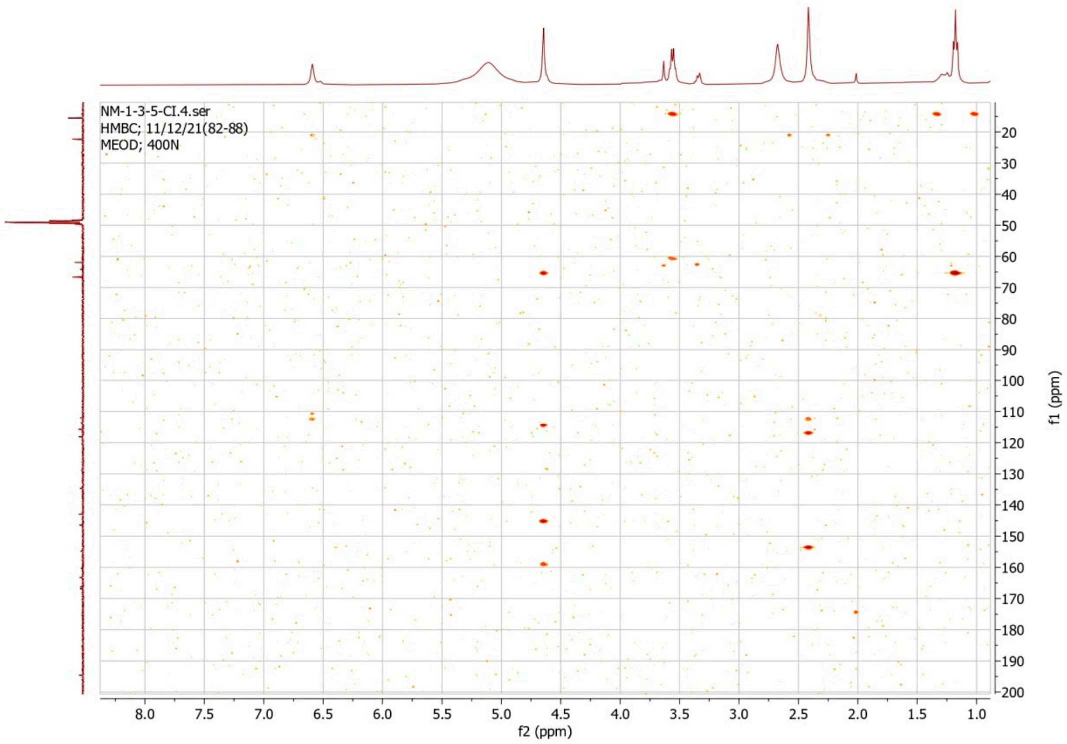

Figure S4. HMBC spectrum of compound **1**

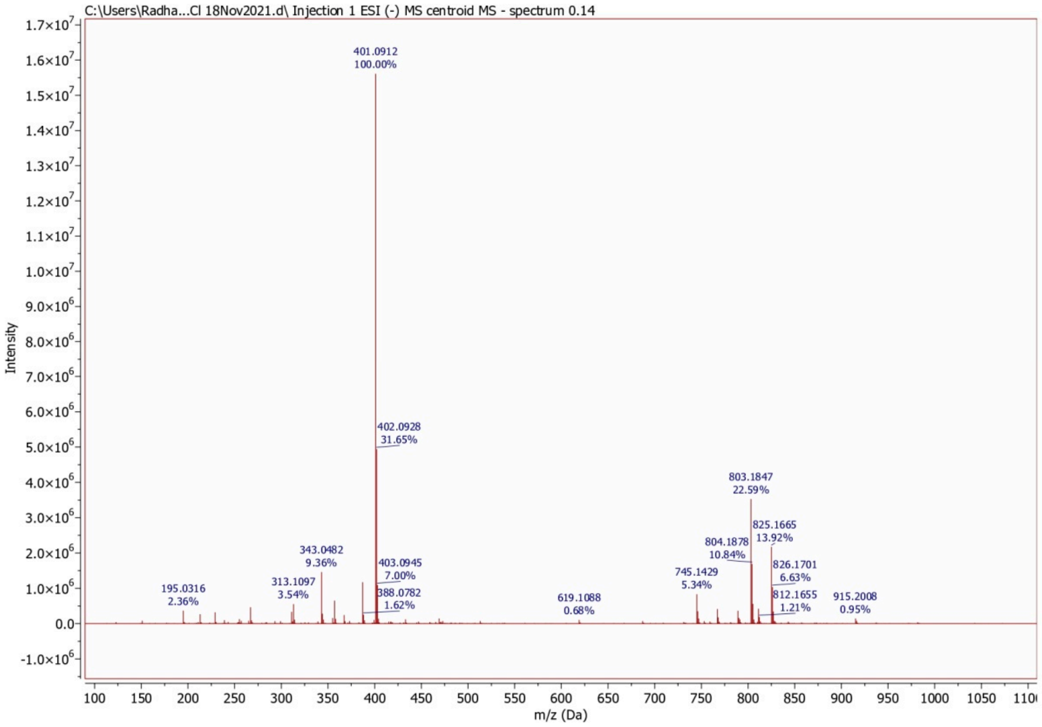

Figure S5. HR-ESI-MS spectrum of compound **1**

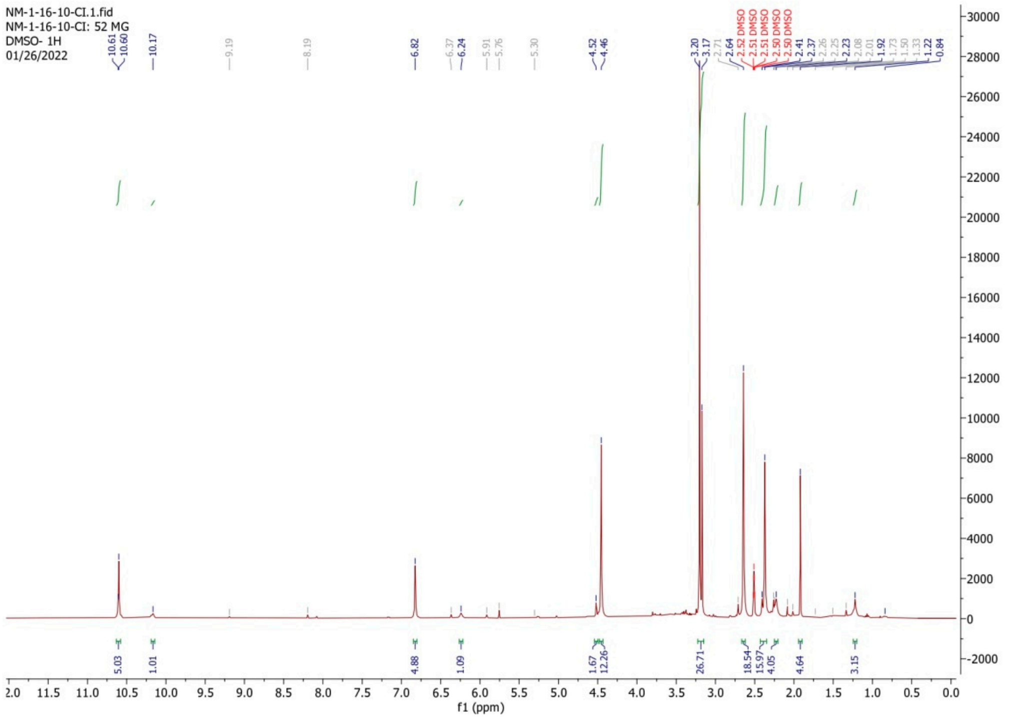

Figure S6. ^{1}H NMR spectrum of compound **2** (400 MHz, DMSO-d_6)

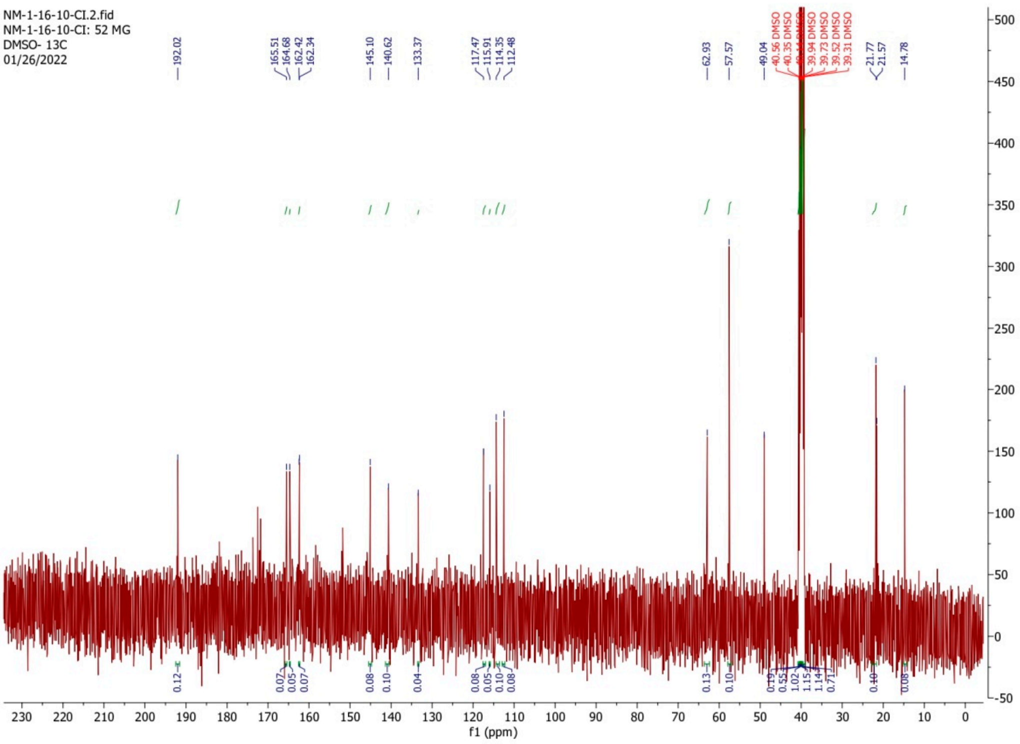

Figure S7. ^{13}C NMR spectrum of compound **2** (100 MHz, DMSO-d_6)

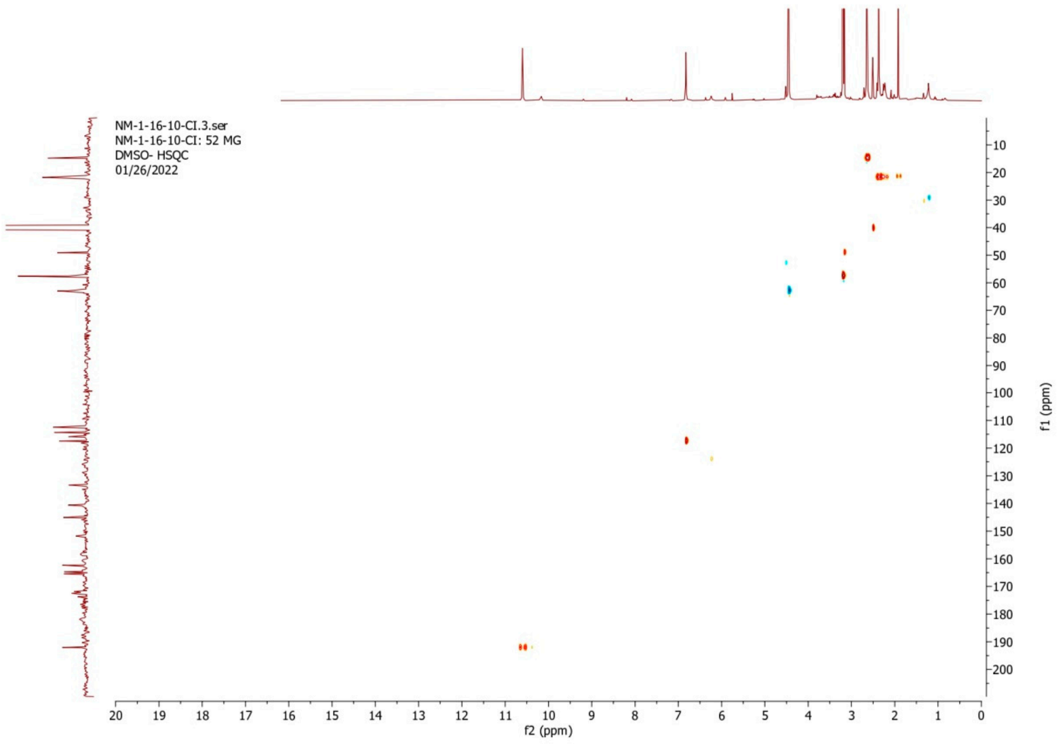

Figure S8. HCQC spectrum of compound **2**

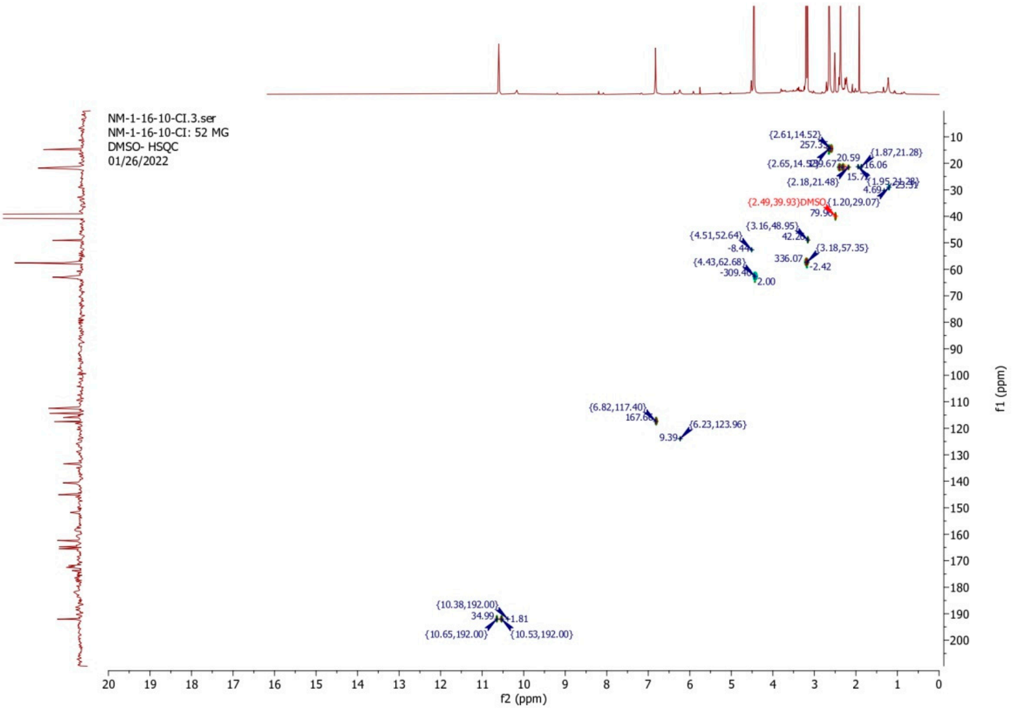

Figure S9. HMBC spectrum of compound **2**

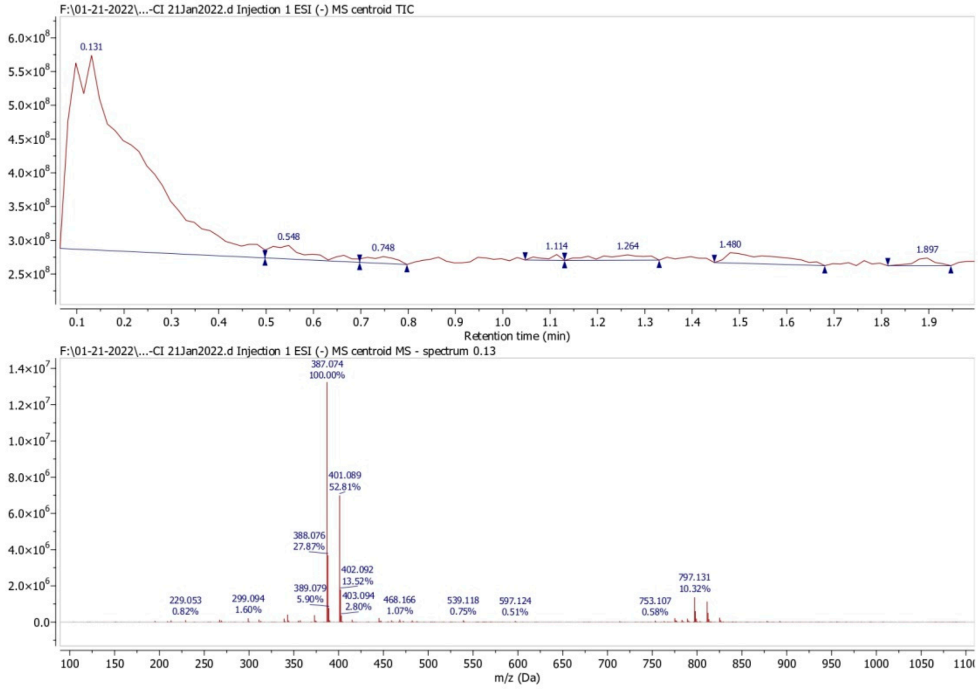

Figure S10. HR-ESI-MS spectrum of compound **2**

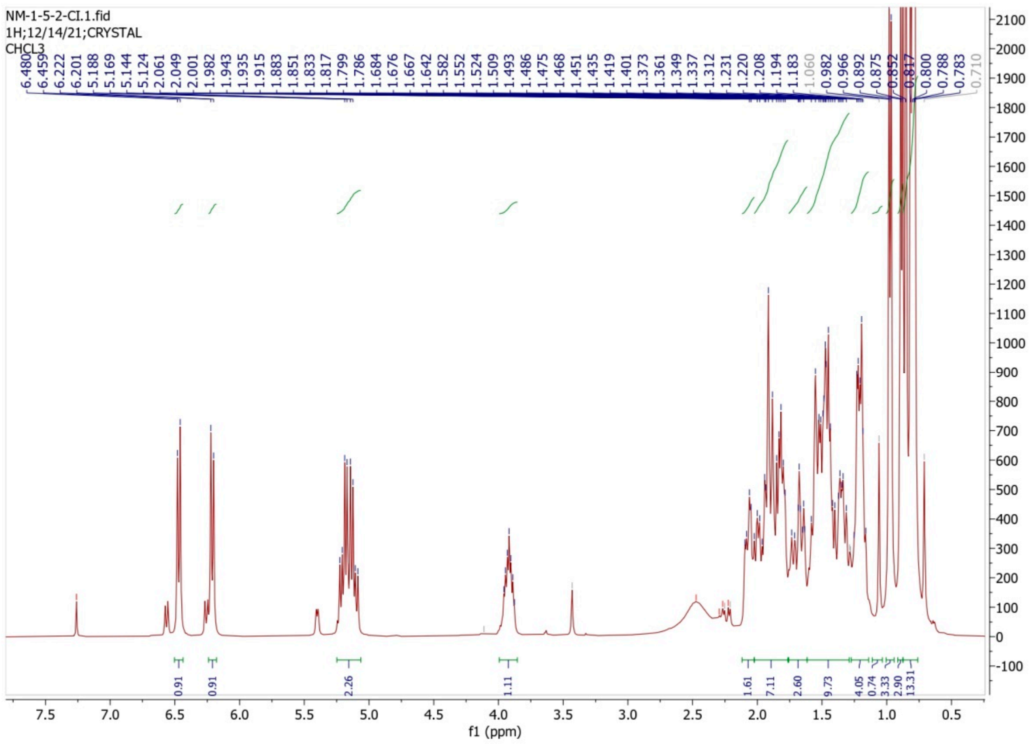

Figure S11. ^{1}H NMR spectrum of compound **4** (400 MHz, $CDCl_3$)

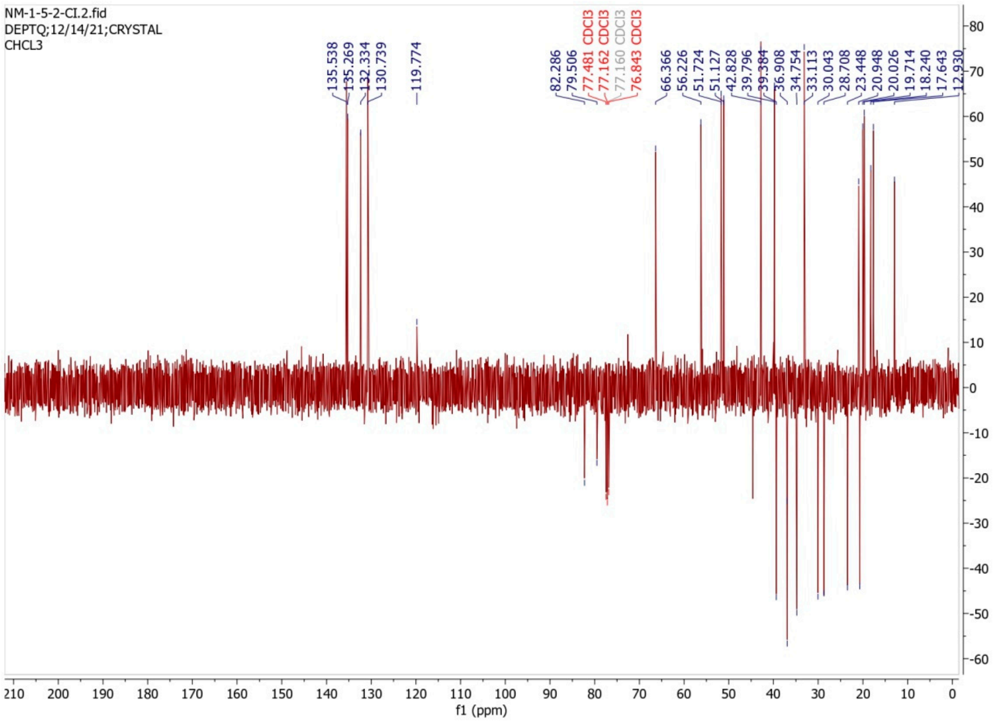

Figure S12. DEPTQ spectrum of compound **4** (100 MHz, $CDCl_3$)

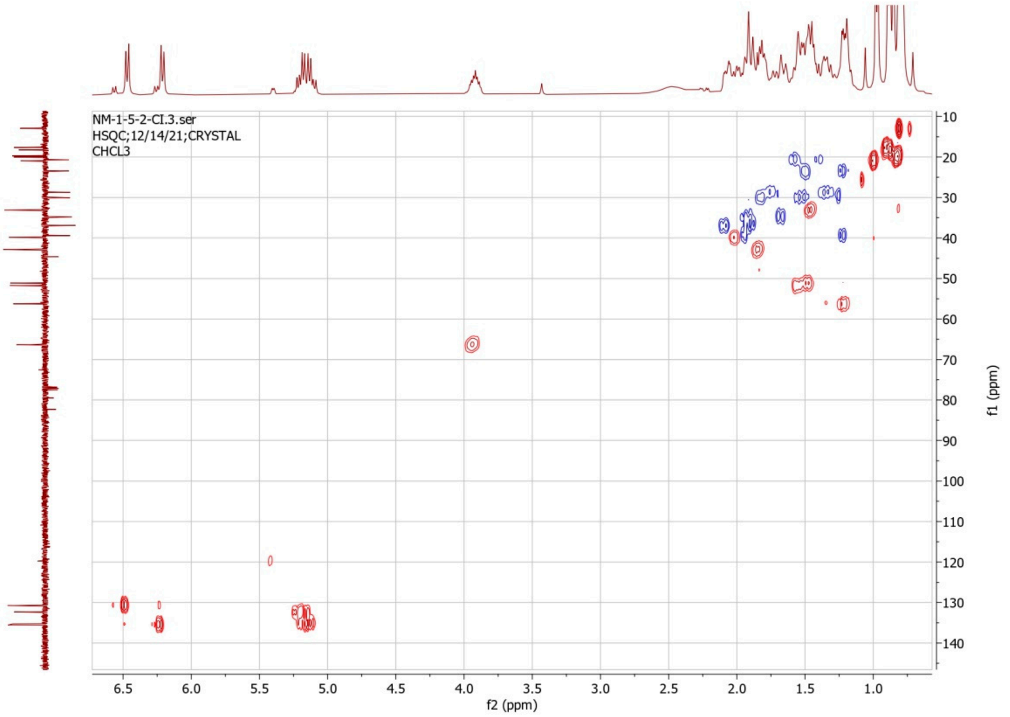

Figure S13. HSQC spectrum of compound **4**

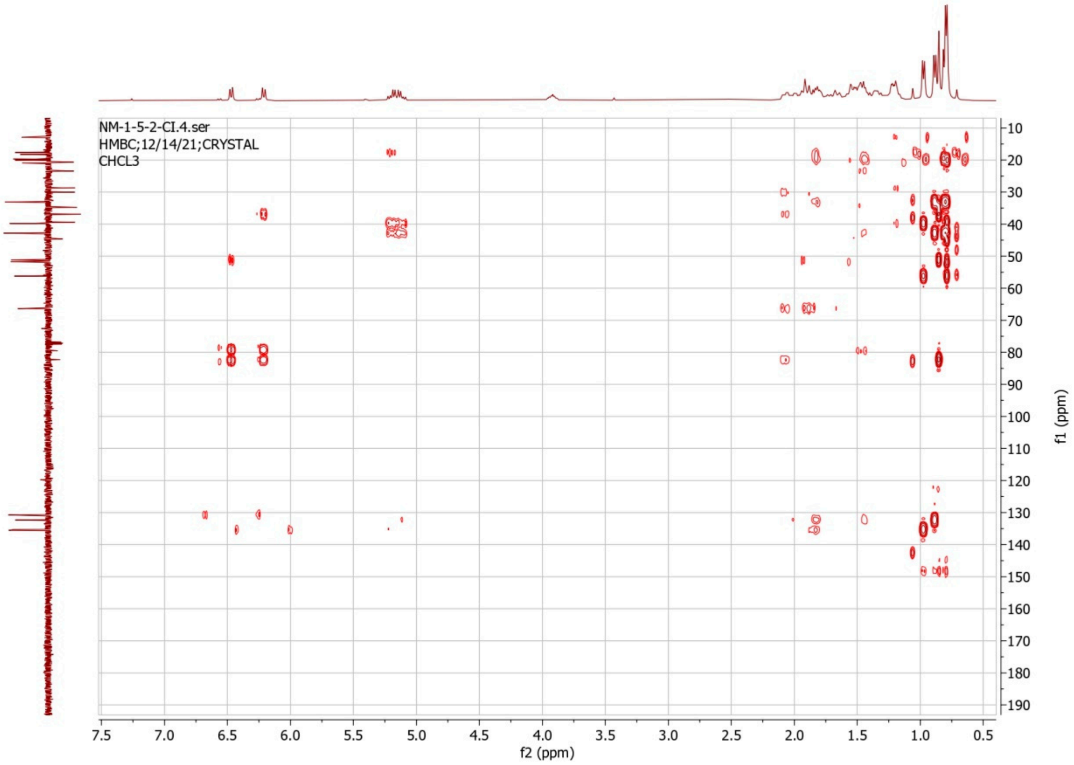

Figure S14. HMBC spectrum of compound **4**

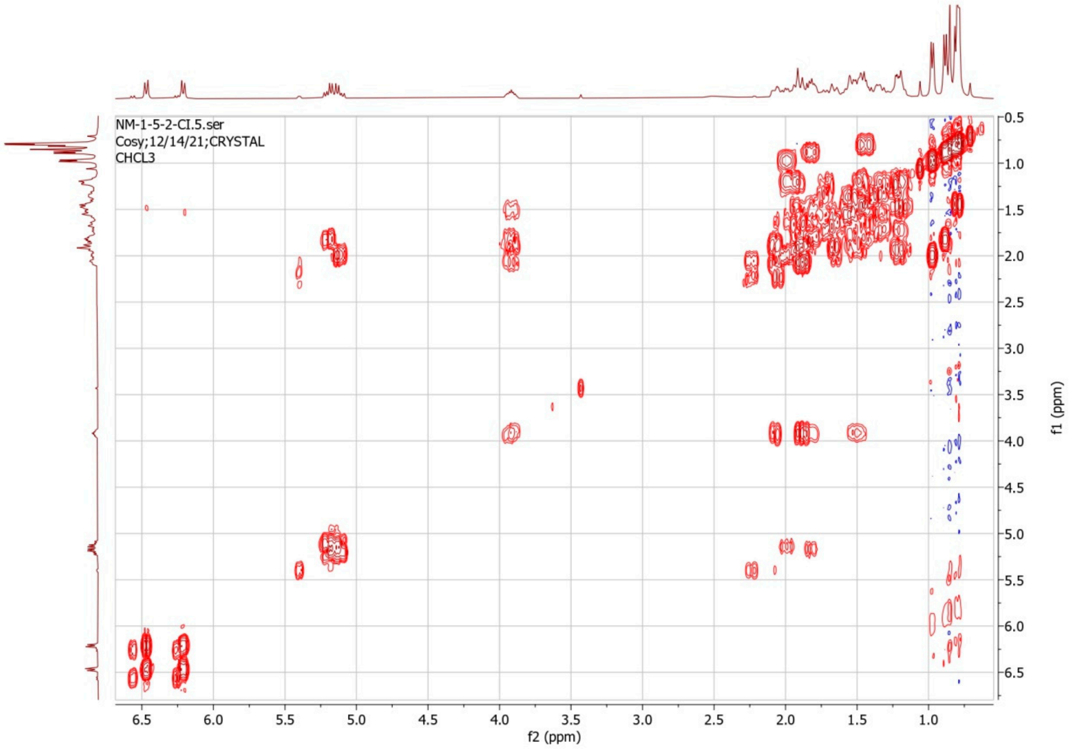

Figure S15. ^{1}H-^{1}H COSY spectrum of compound **4**

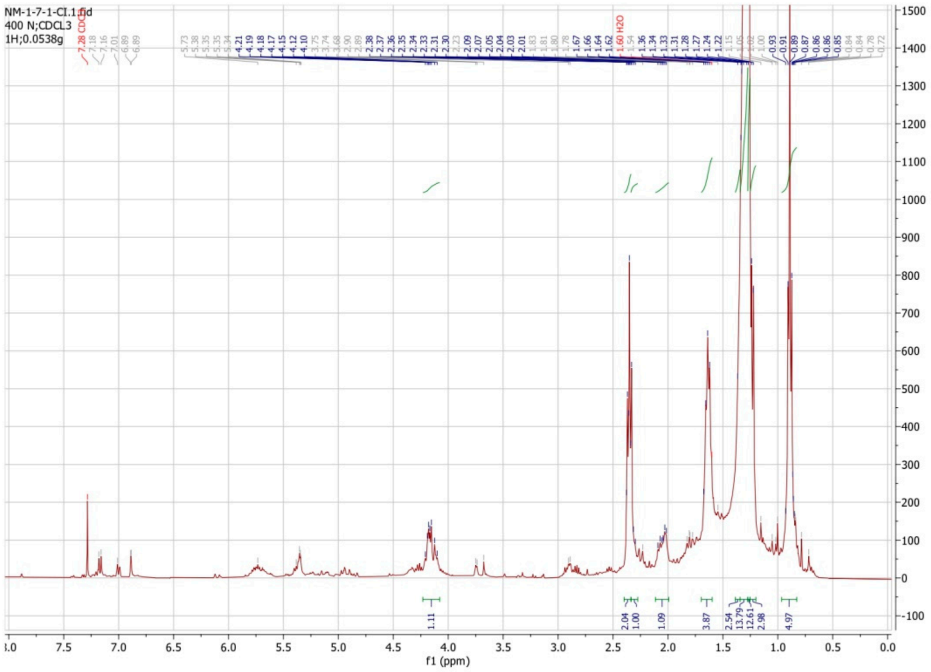

Figure S16. ^{1}H NMR spectrum of mixture of compounds **5,6,7** (400 MHz, $CDCl_3$)

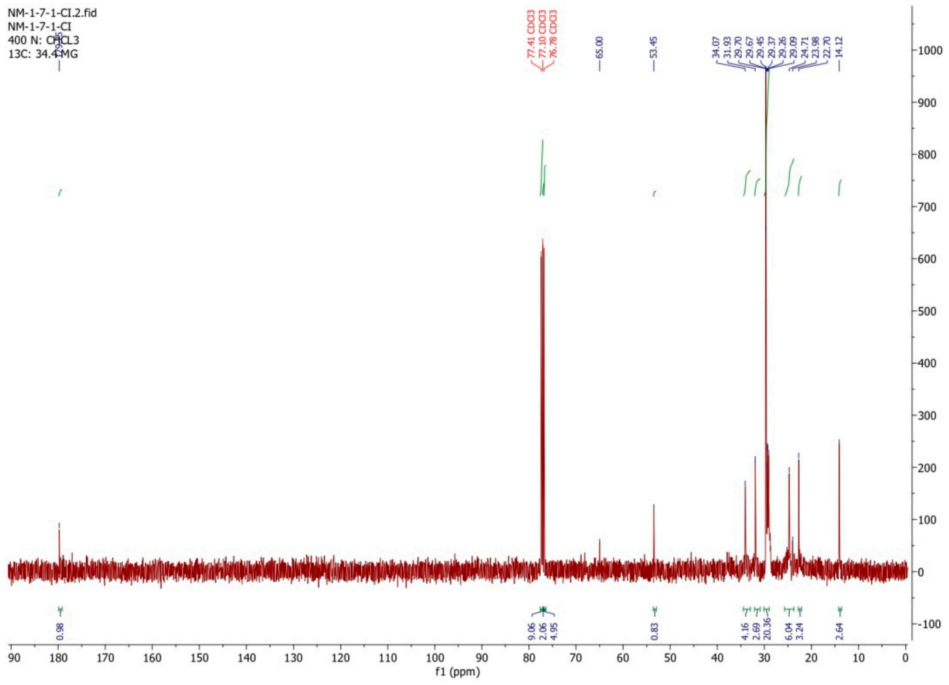

Figure S17. ^{13}C NMR spectrum of mixture of compounds **5,6,7** (100 MHz, $CDCl_3$)

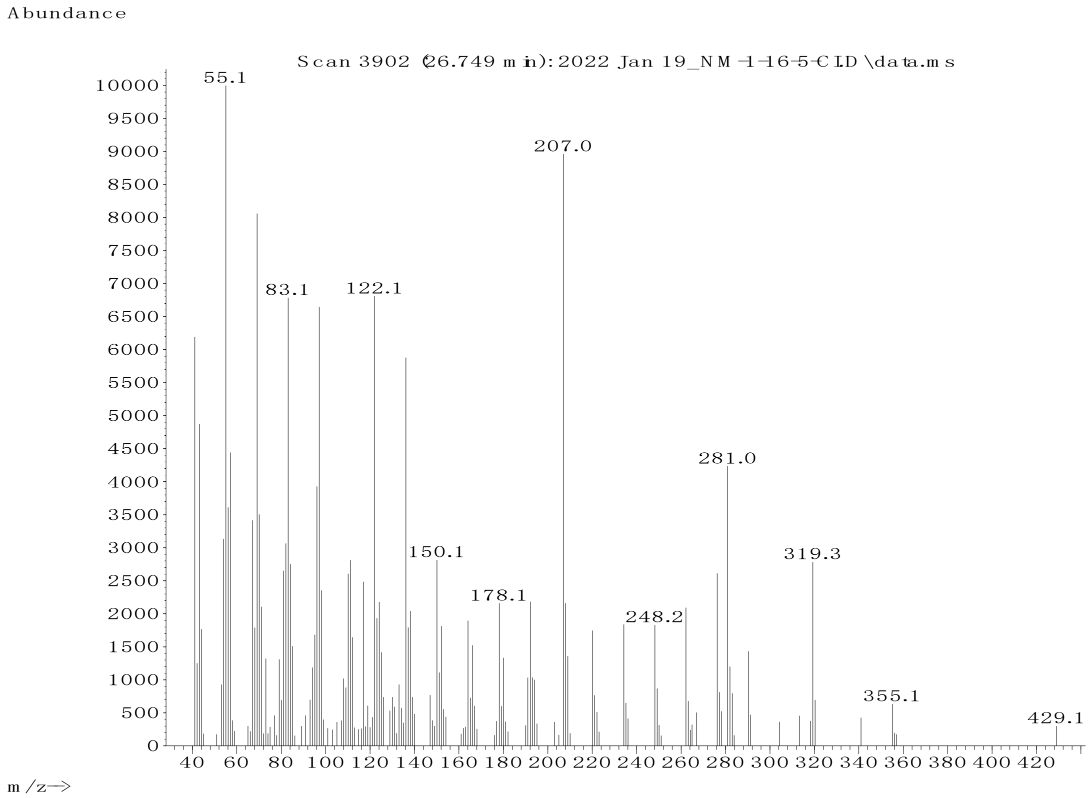

Figure S18. EI-MS spectrum of compound **5**

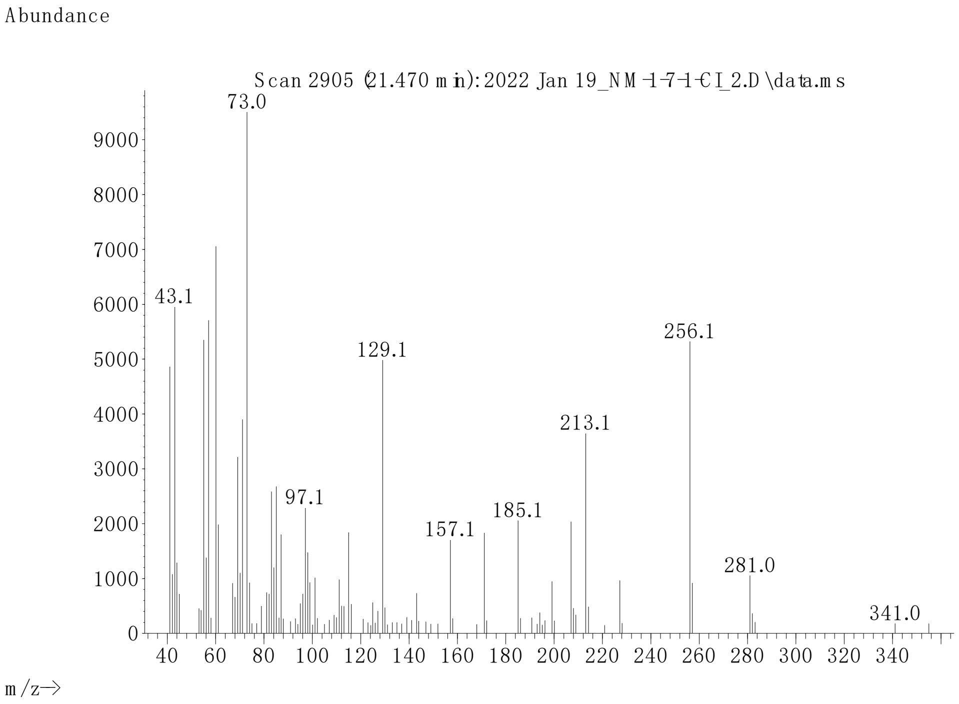

Figure S19. EI-MS spectrum of compound **6**

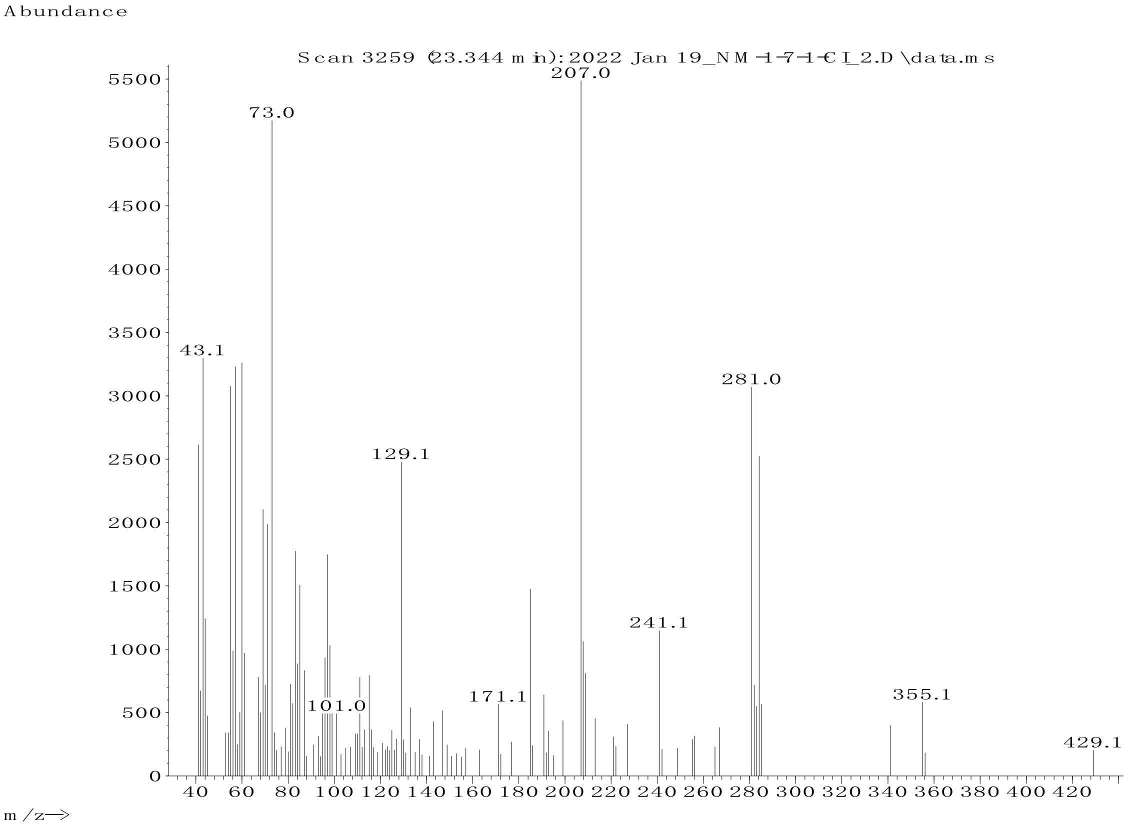

Figure S20. EI-MS spectrum of compound **7**

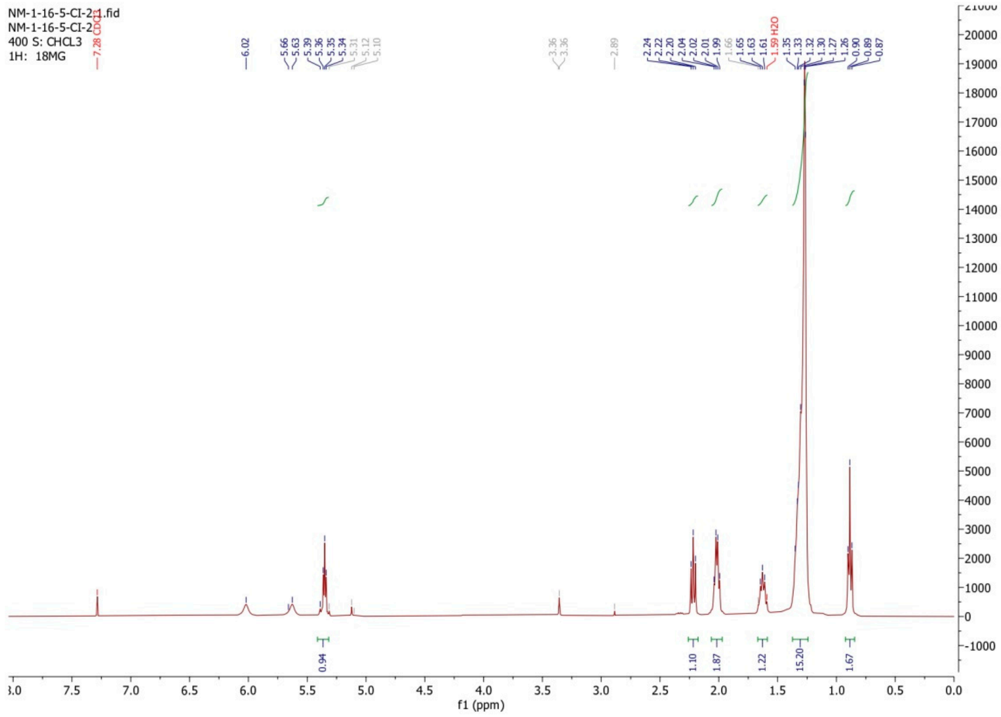

Figure S21. ^{1}H NMR spectrum of mixture of compounds **5** (400 MHz, $CDCl_3$)

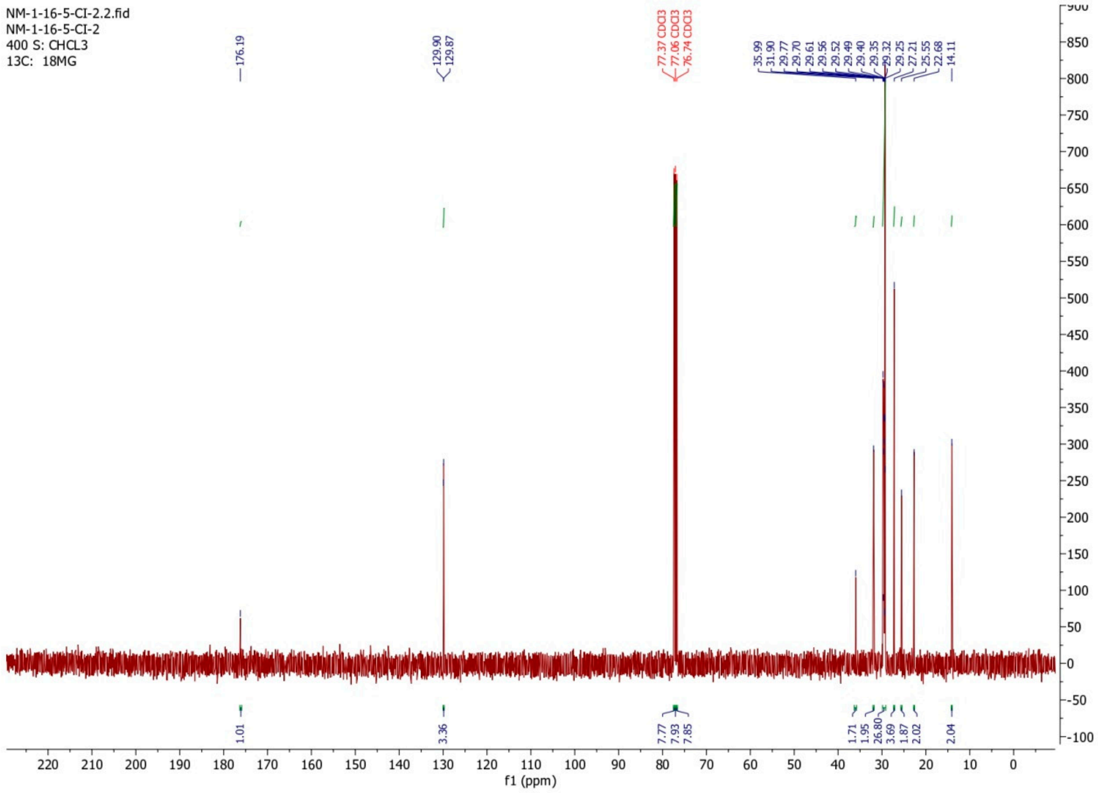

Figure S22. ^{13}C NMR spectrum of mixture of compounds **5** (100 MHz, $CDCl_3$)

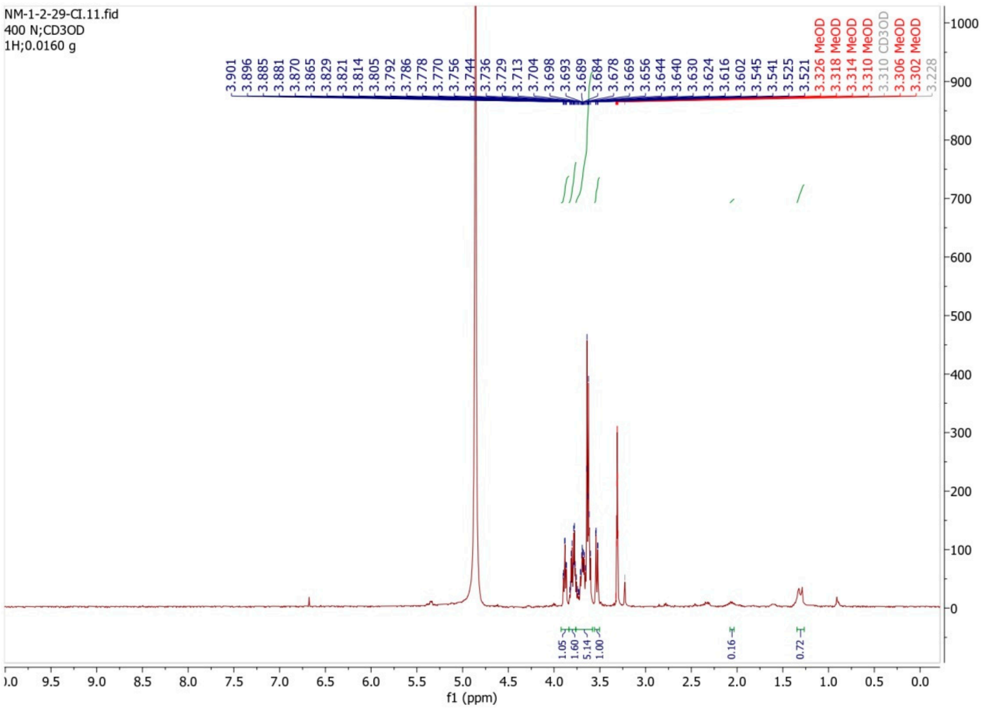

Figure S23. ^{1}H NMR spectrum of mixture of compounds **8** (400 MHz, CD_3OD)

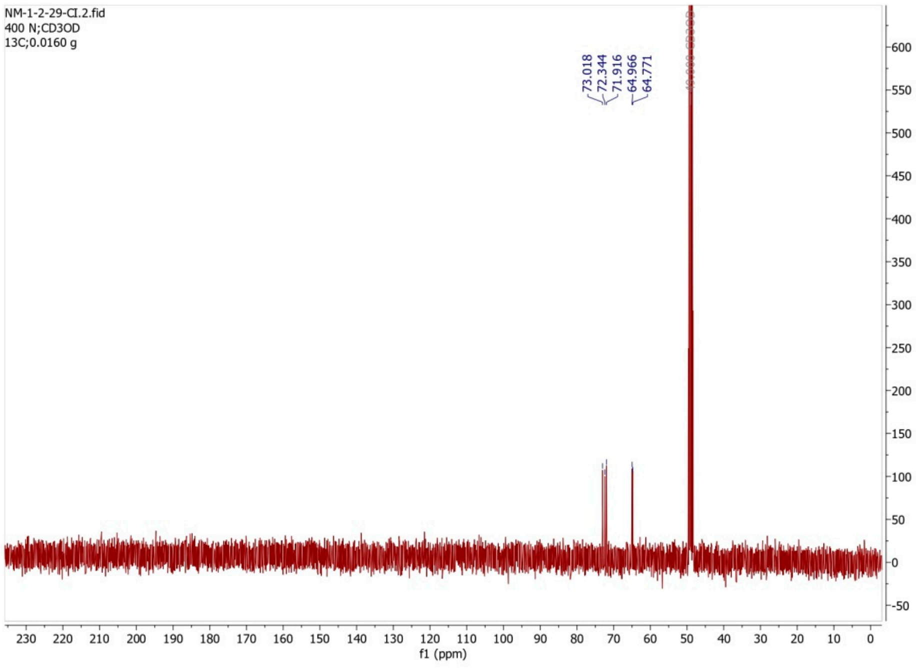

Figure S24. ^{13}C NMR spectrum of mixture of compounds **8** (100 MHz, CD_3OD)

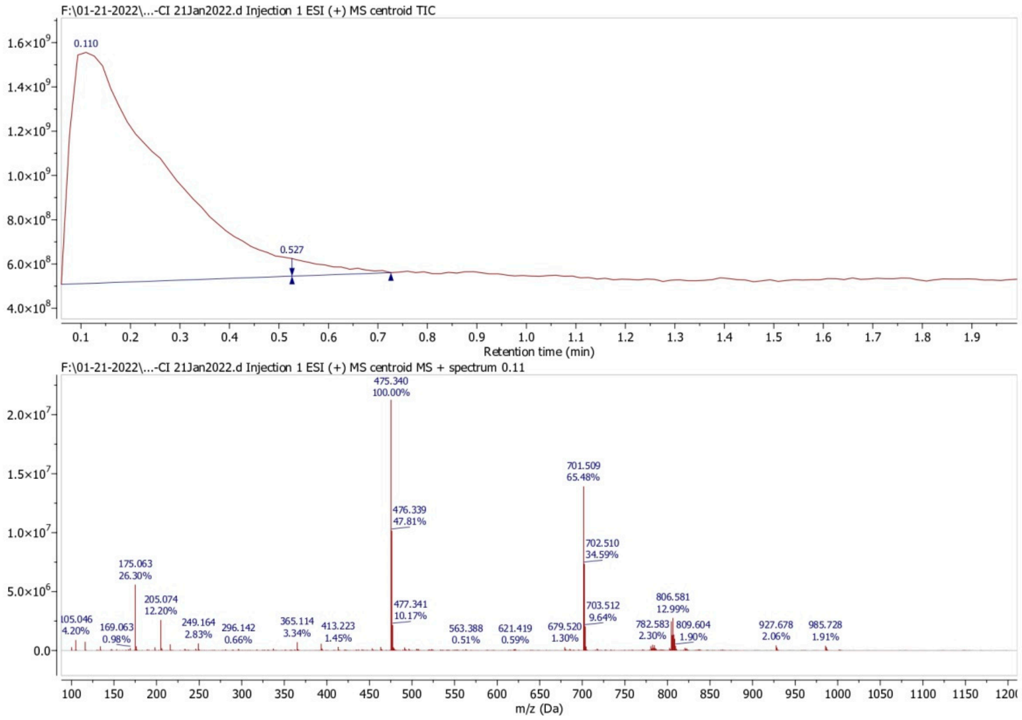

Figure S25. HR-ESI-MS spectrum of compound **8**

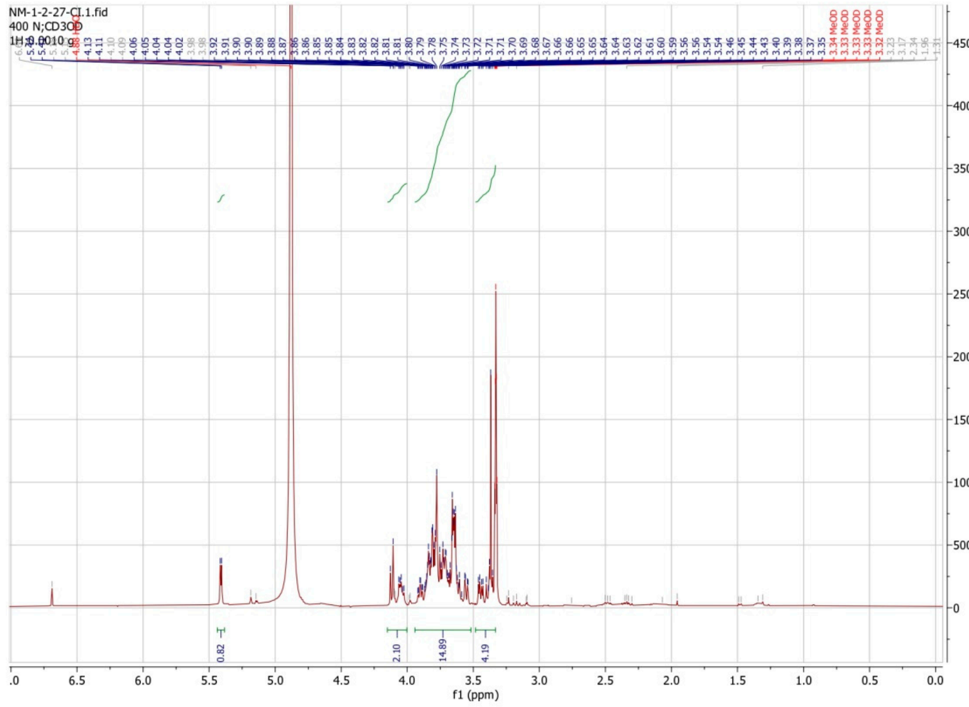

Figure S26. ^{1}H NMR spectrum of mixture of compounds **9** (400 MHz, CD_3OD)

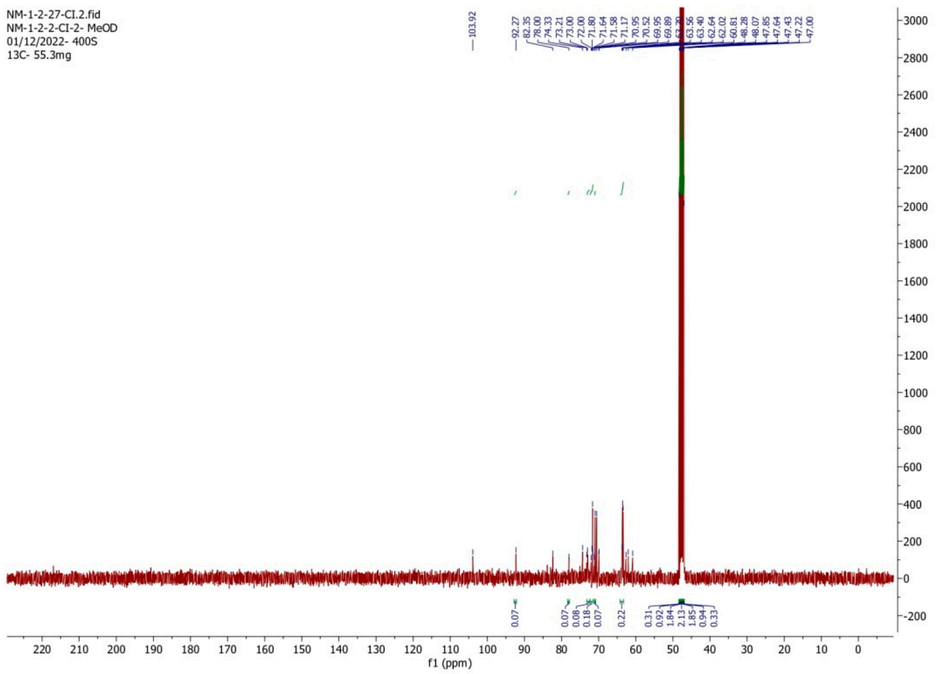

Figure S27. ^{13}C NMR spectrum of mixture of compounds **9** (100 MHz, CD_3OD)

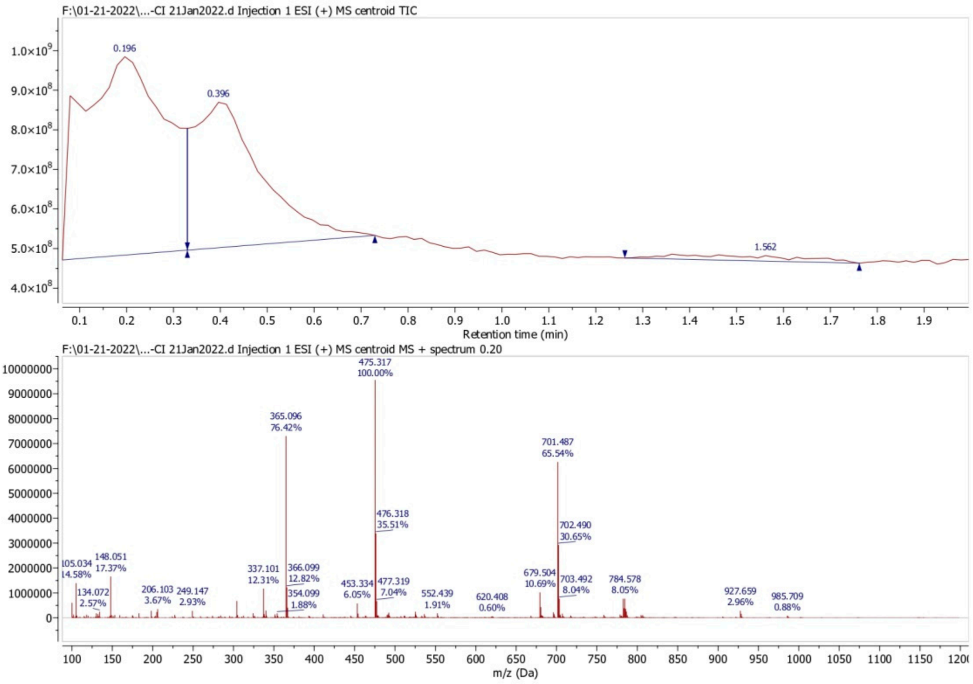

Figure S28. HR-ESI-MS spectrum of compound **9**

Peak-1

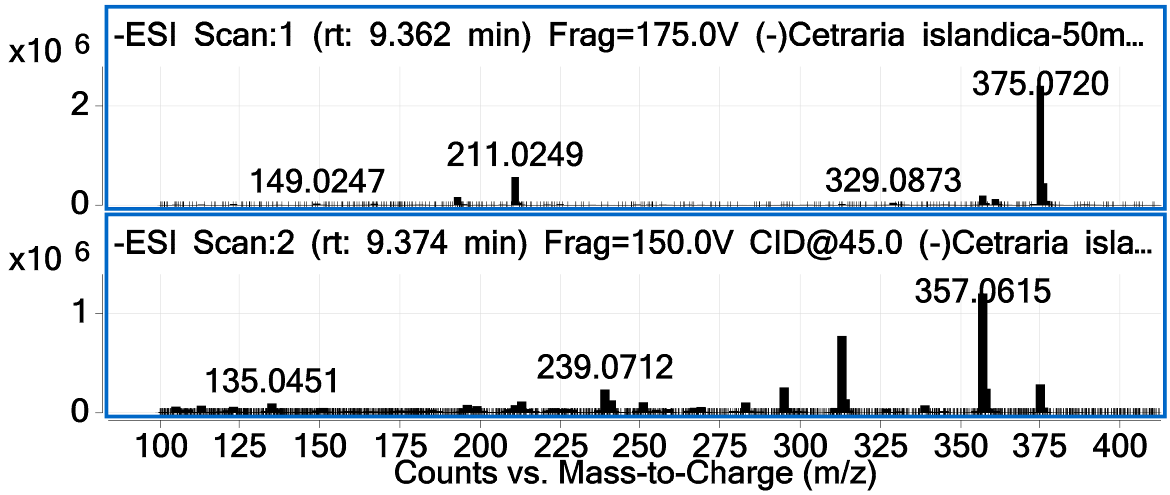

Peak-2

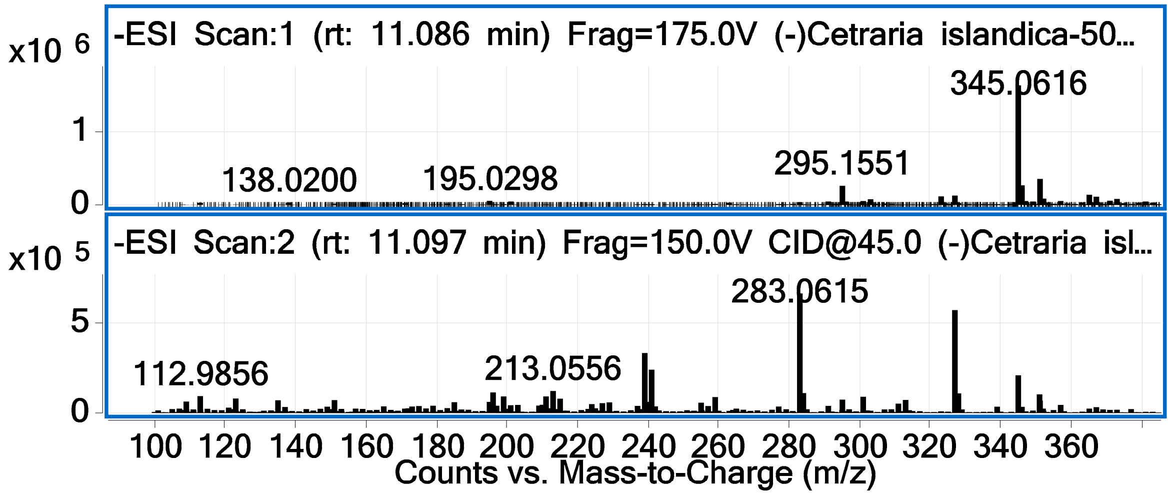

Peak-3

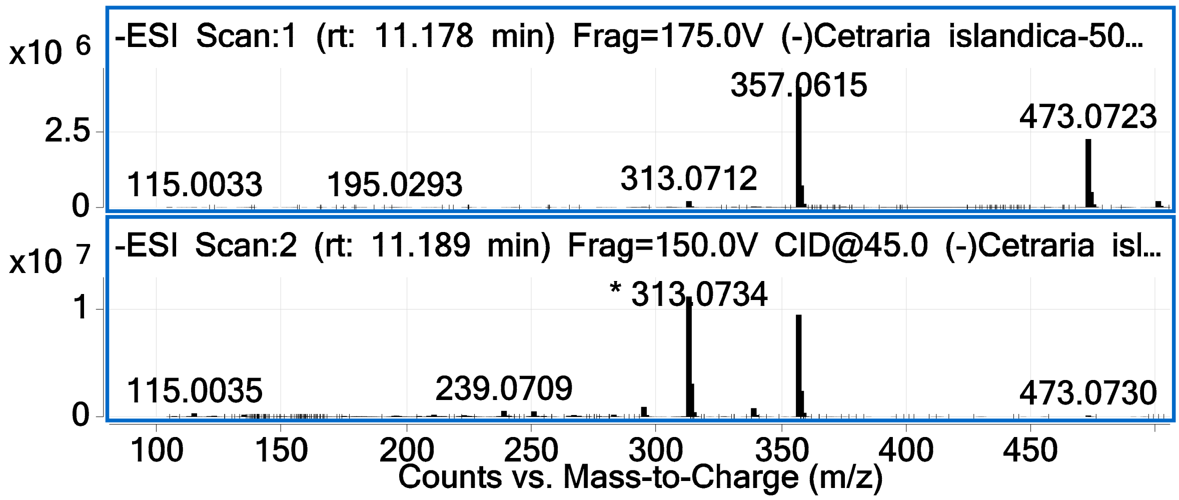

Peak-4

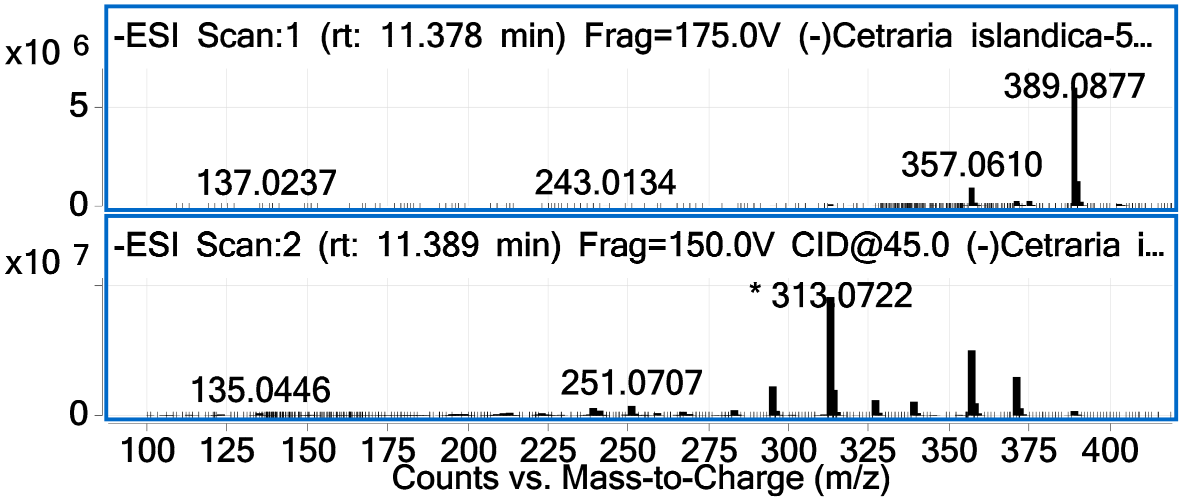

Peak-5

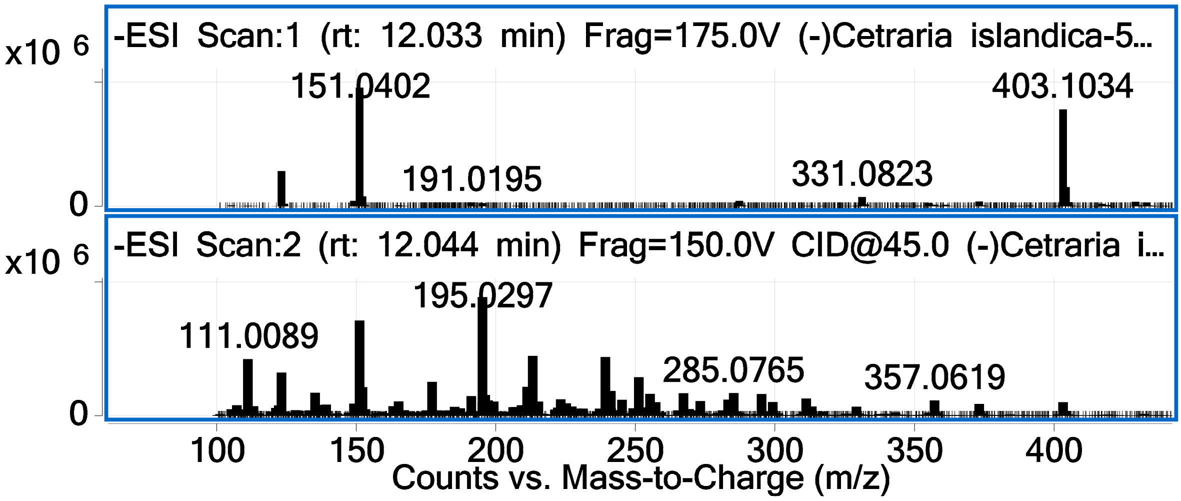

Peak-6

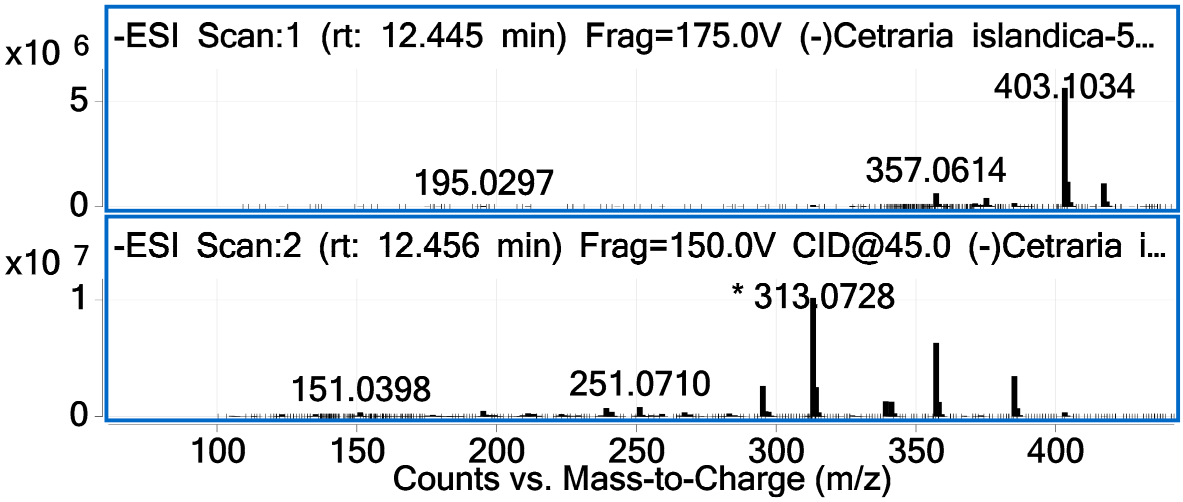

Peak-7

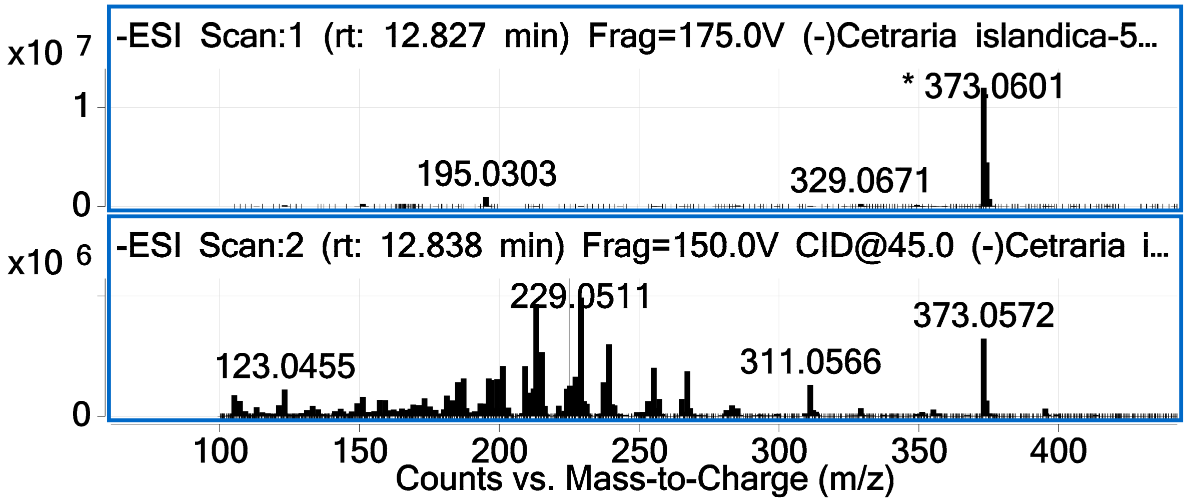

Peak-8

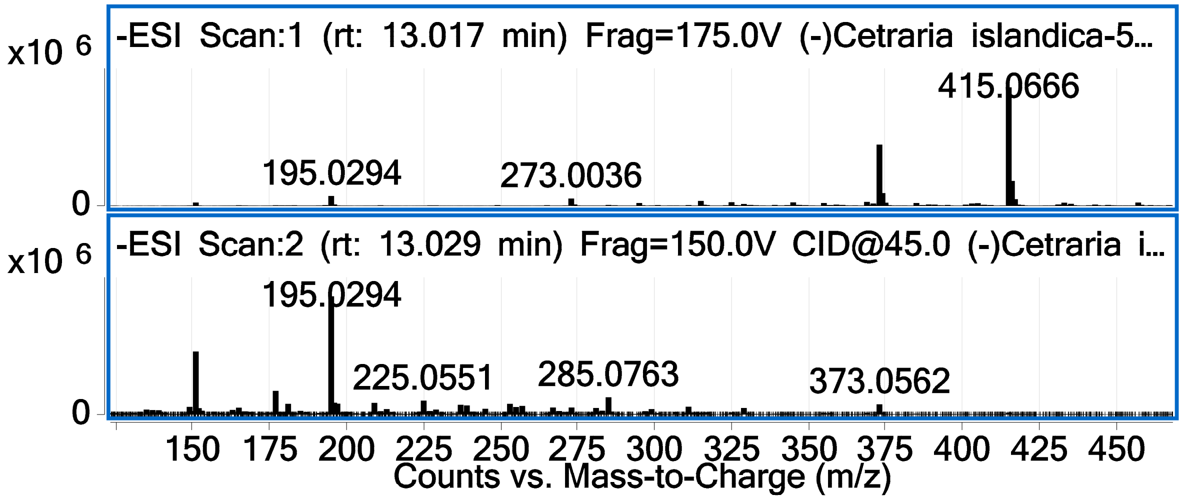

Peak-9

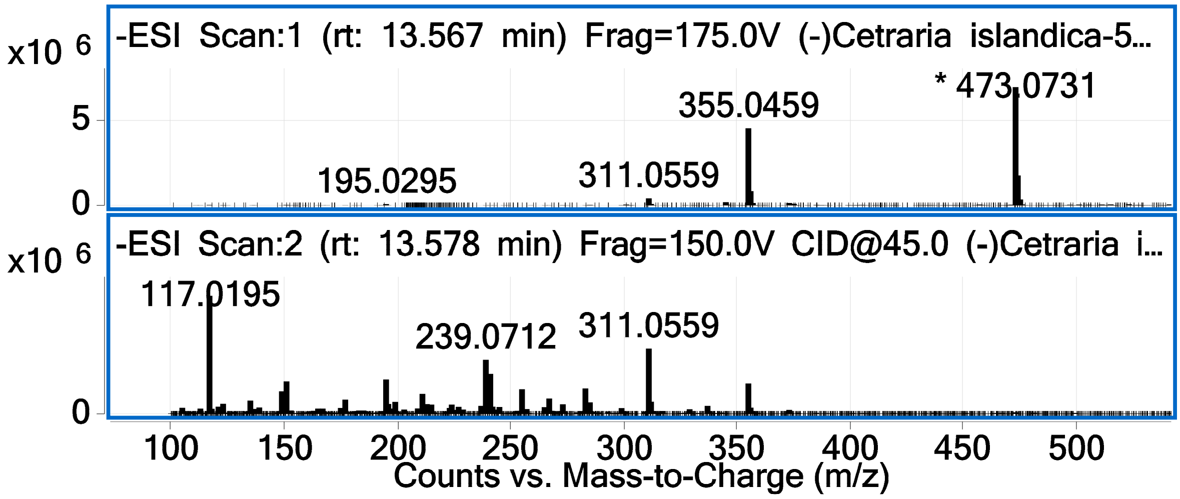

Peak-10

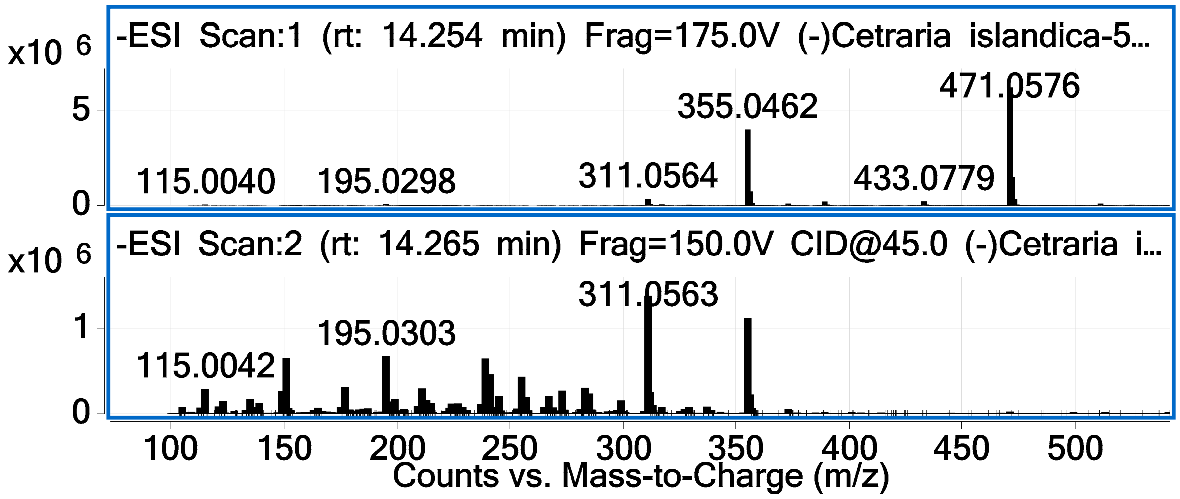

Peak-11

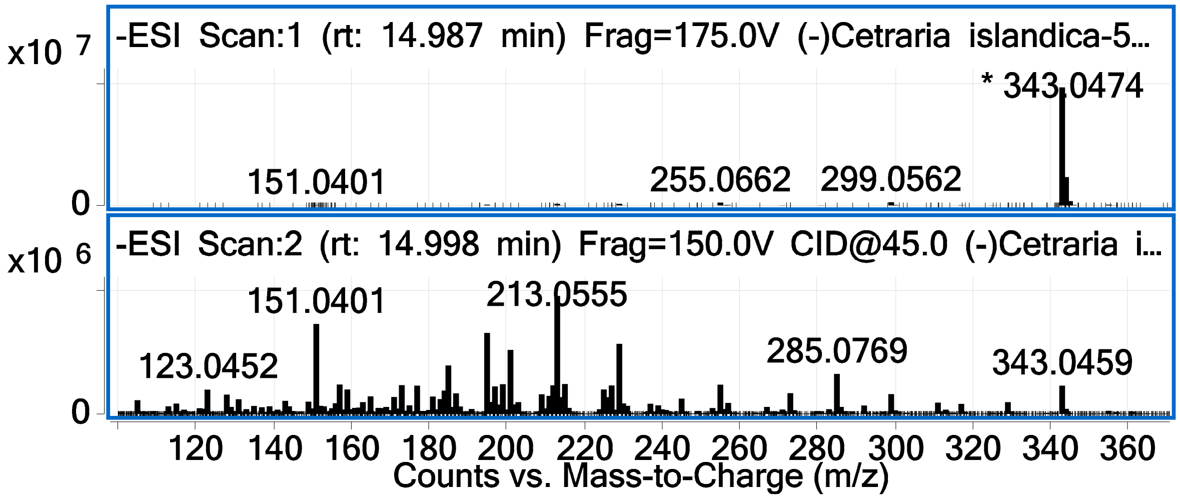

Peak-12

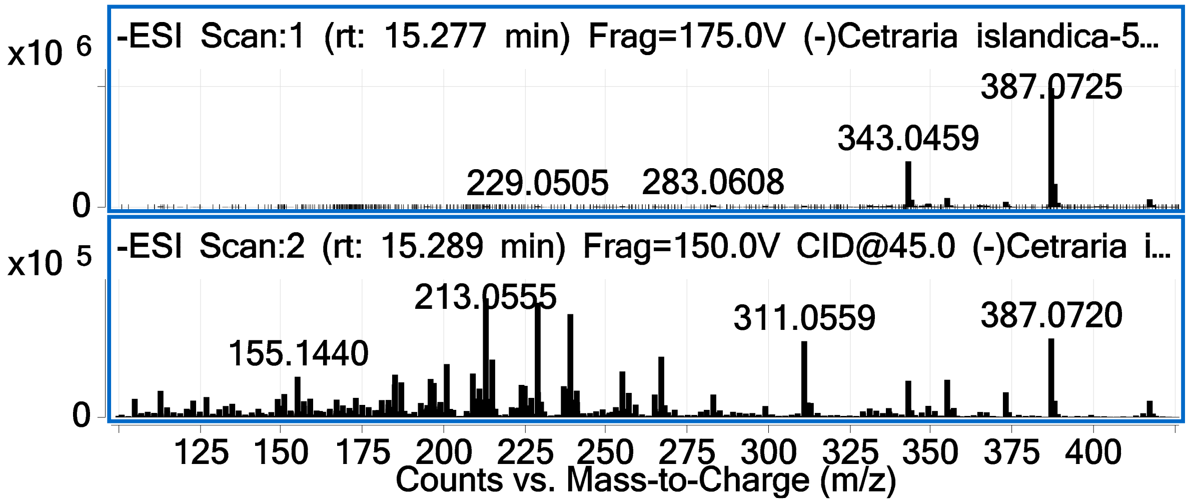

Peak-13

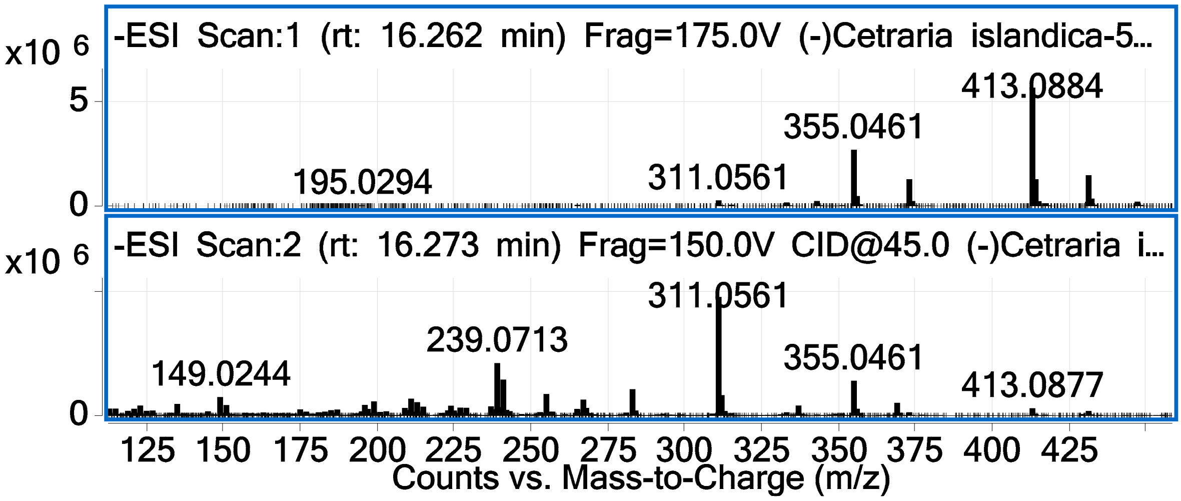

Peak-14

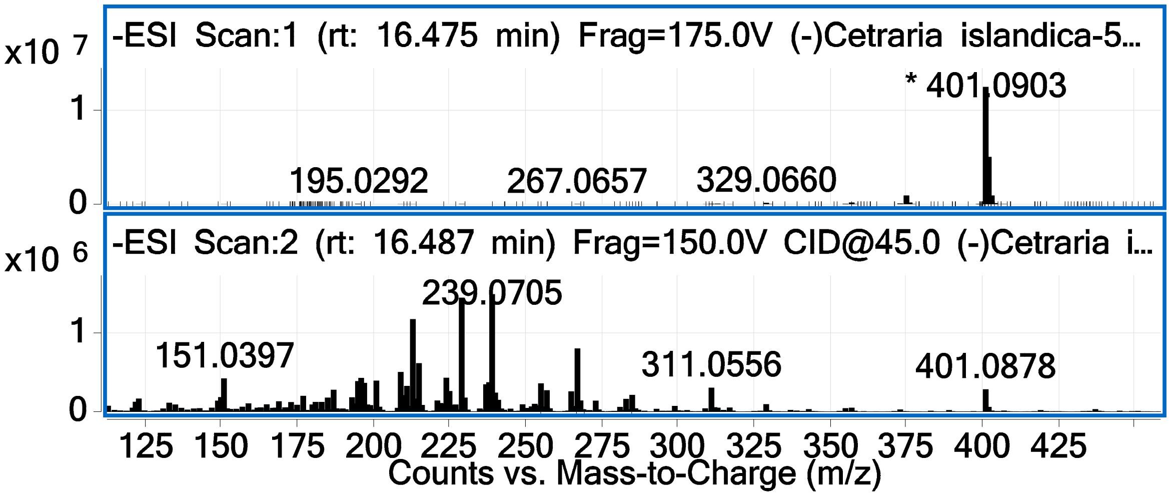

Peak-15

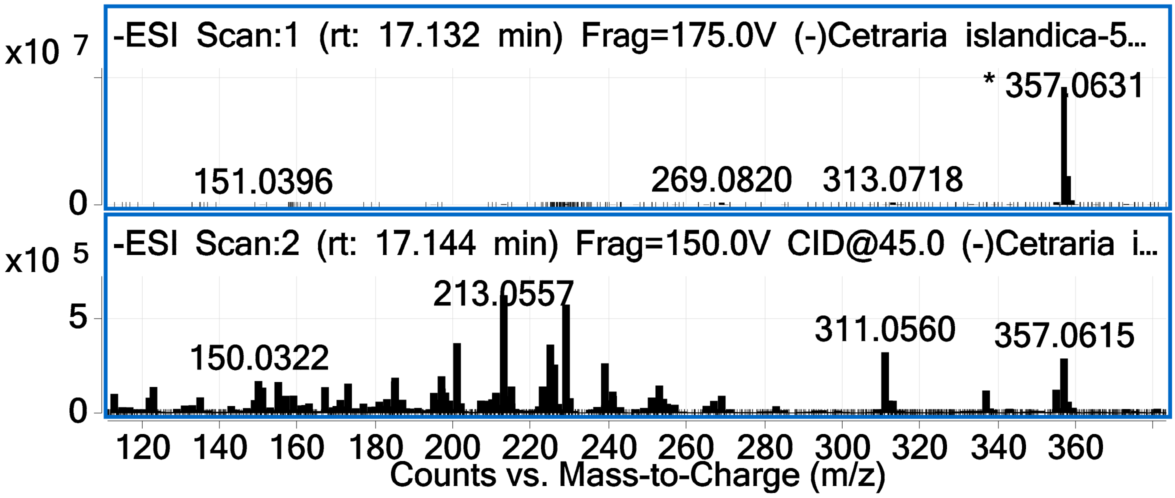

Peak-16

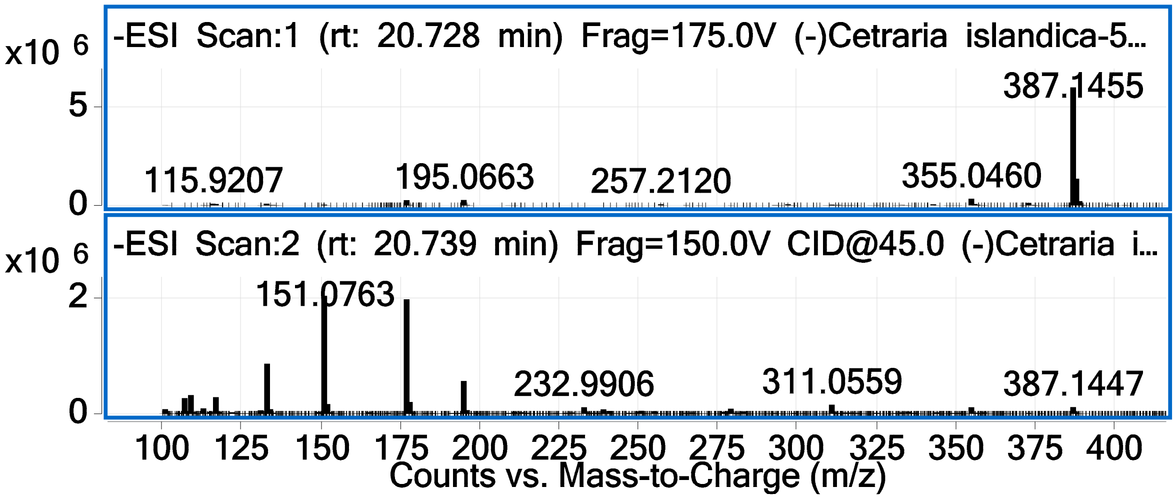

Peak-17

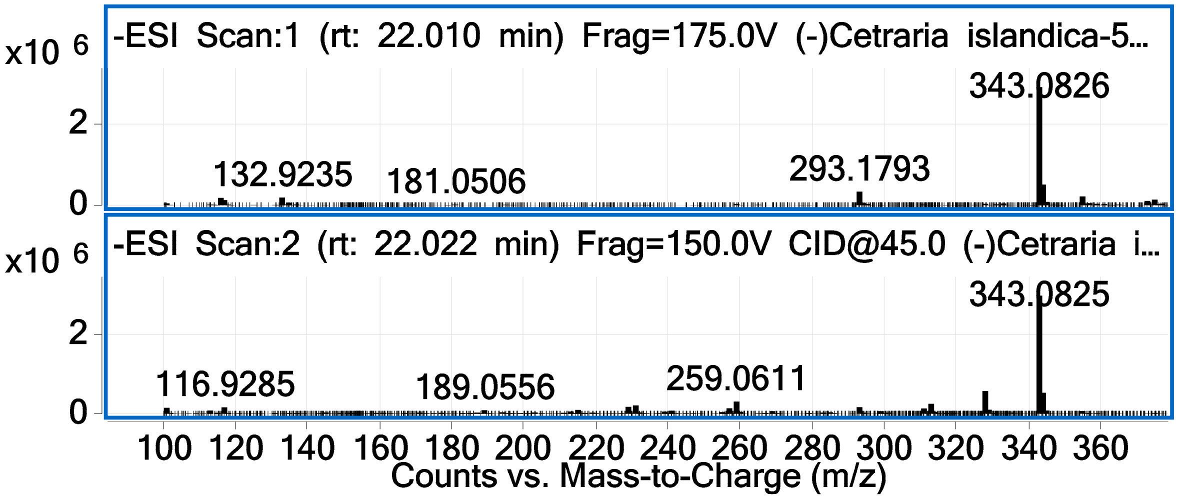

Peak-18

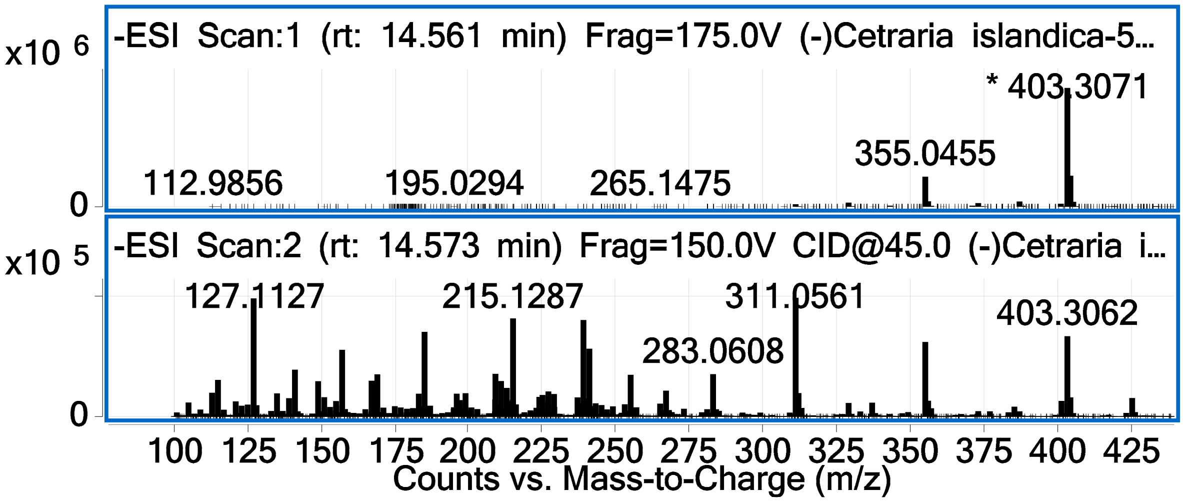

Peak-19

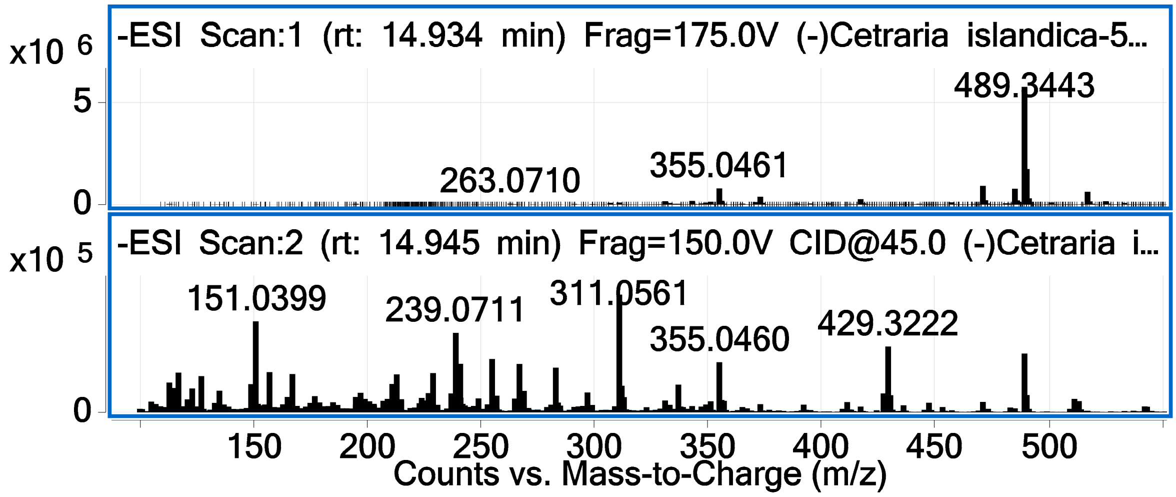

Peak-20

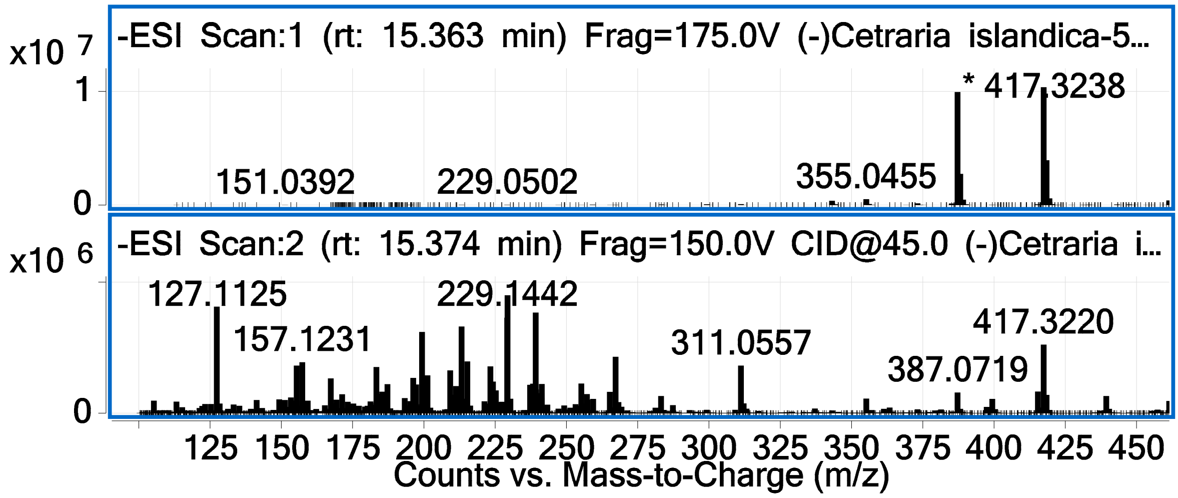

Peak-21

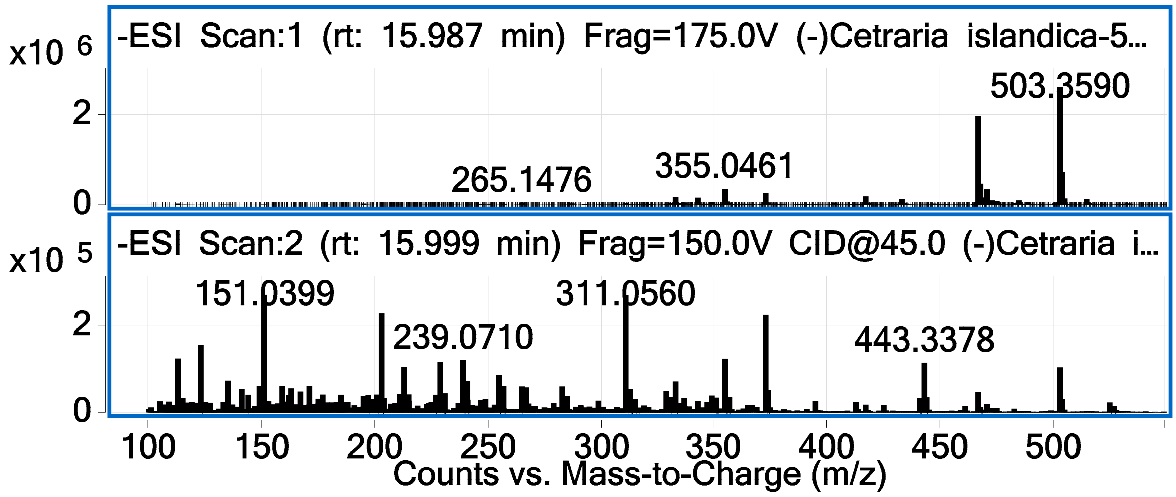

Peak-22

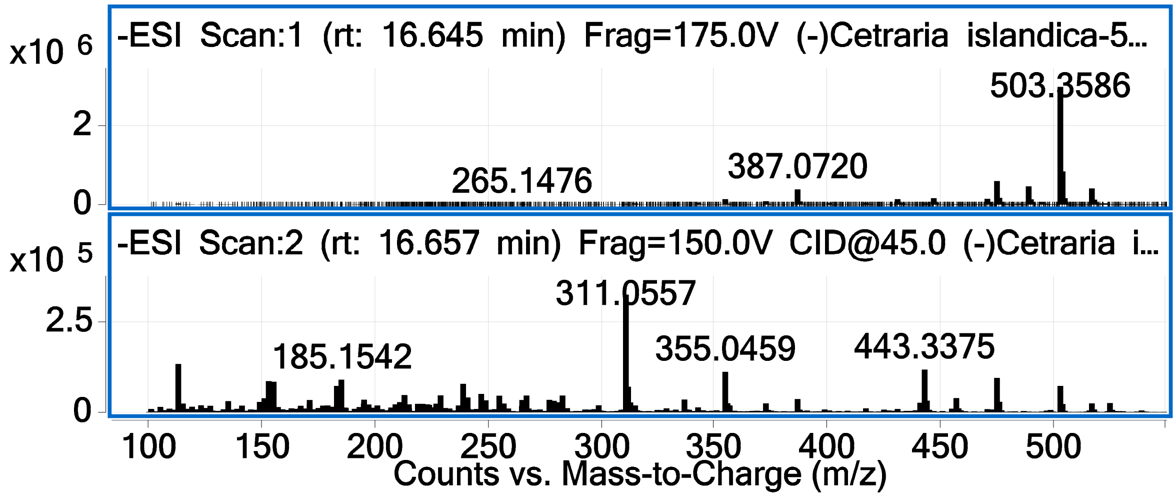

Peak-23

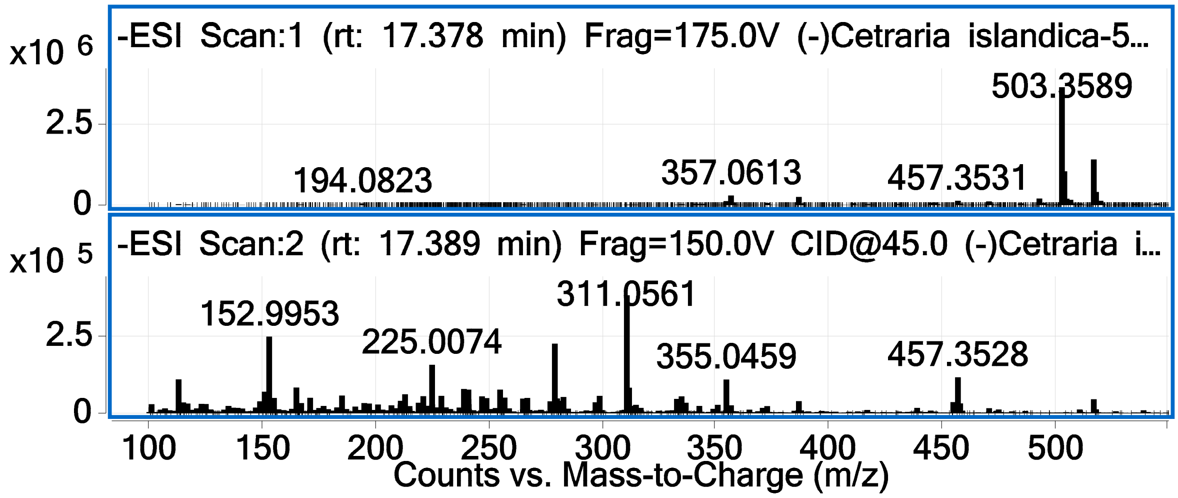

Peak-24

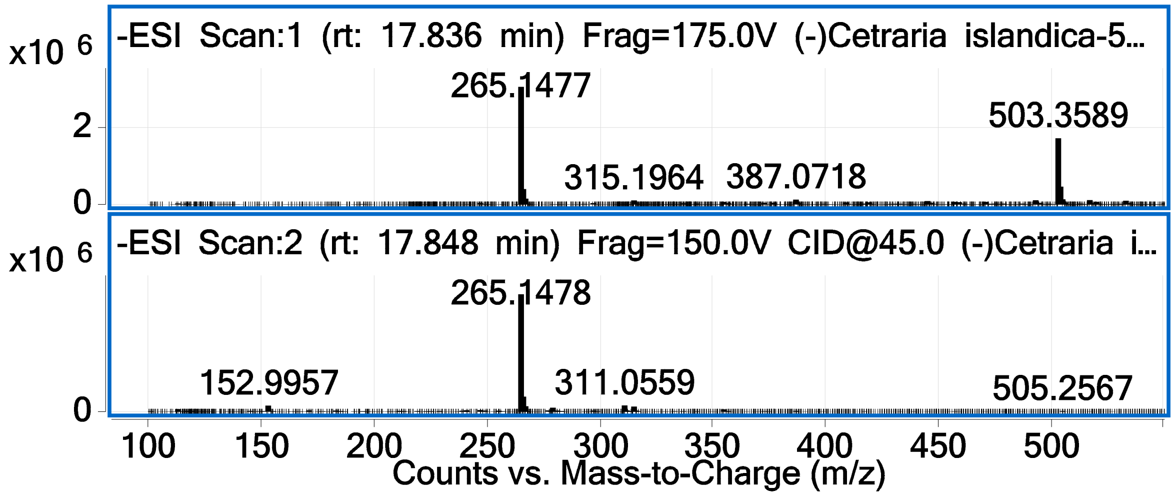

Peak-25

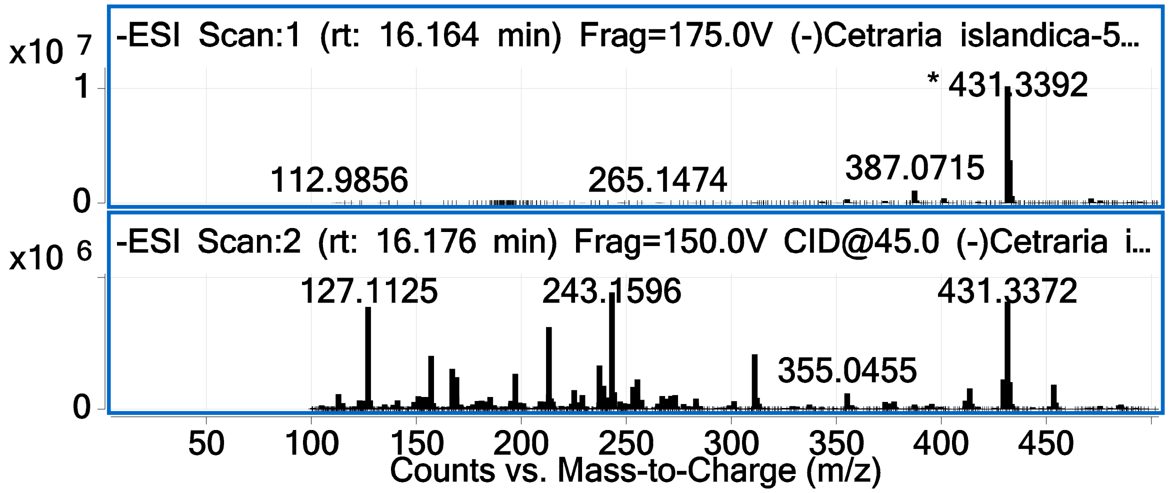

<u>Peak-26</u>

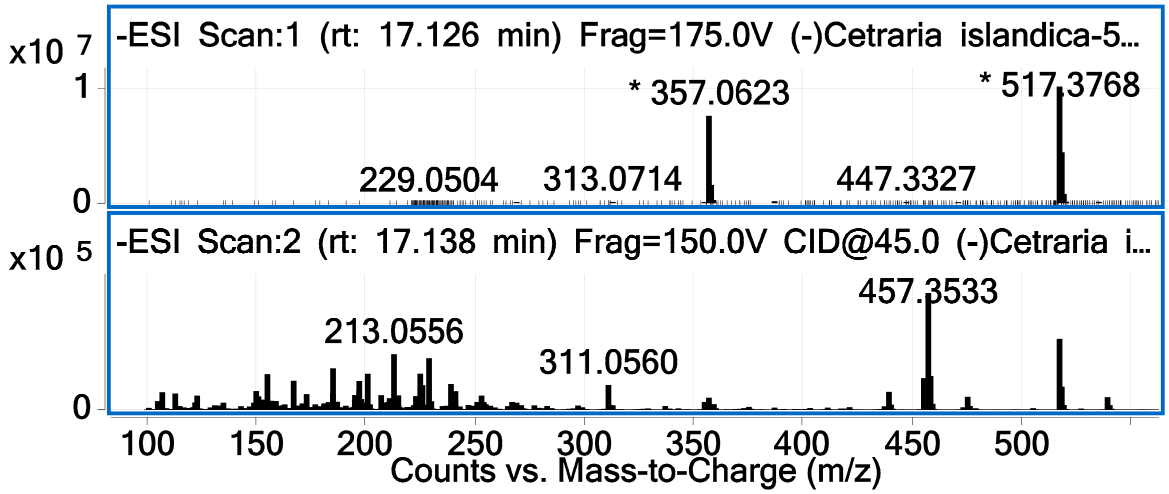

<u>Peak-27</u>

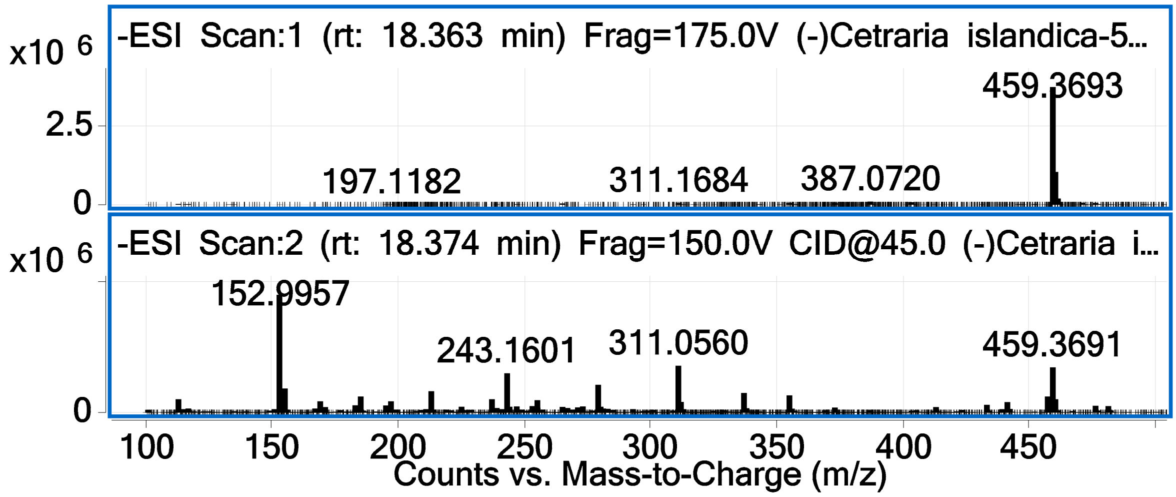

<u>Peak-28</u>

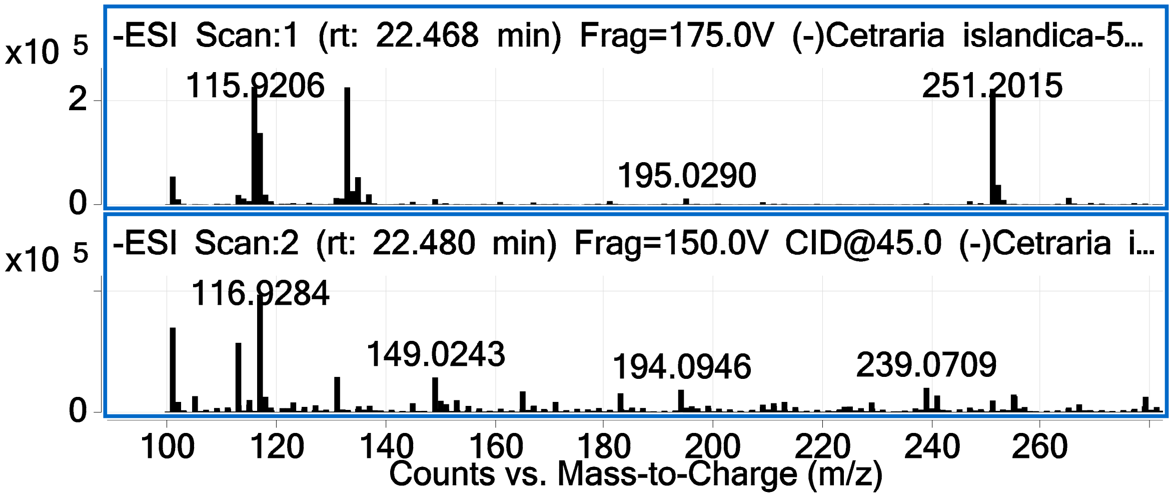

Peak-29

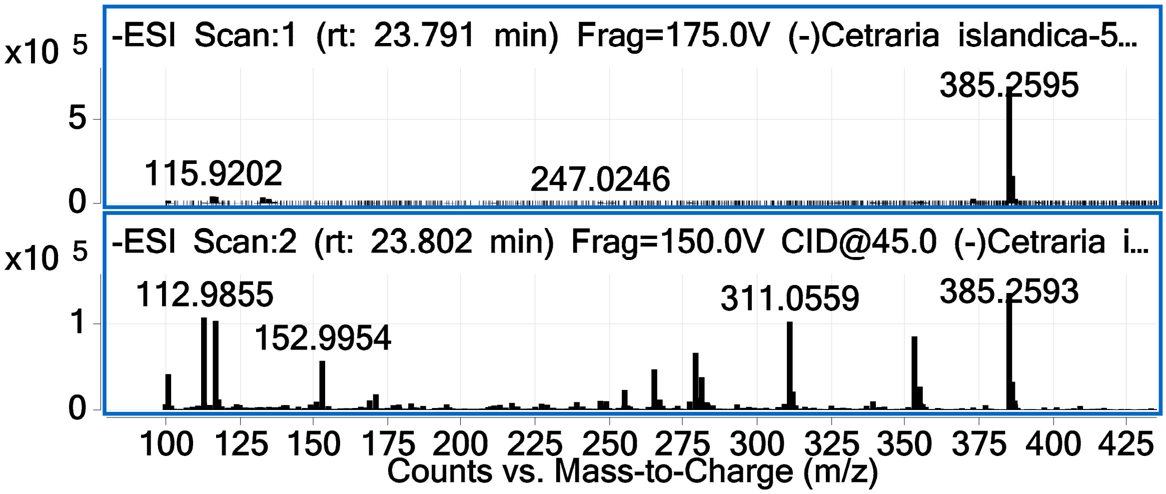

Peak-30

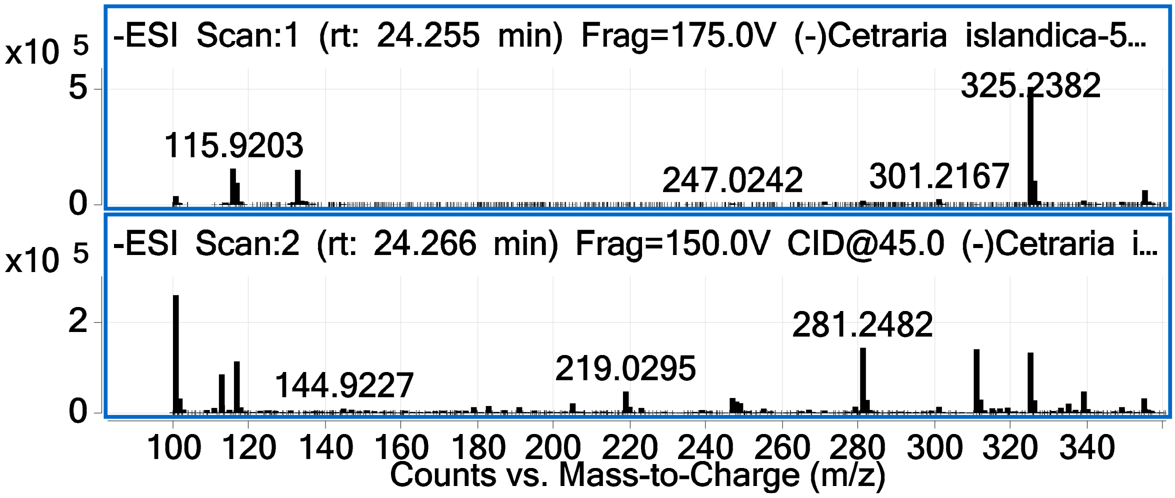

Peak-31

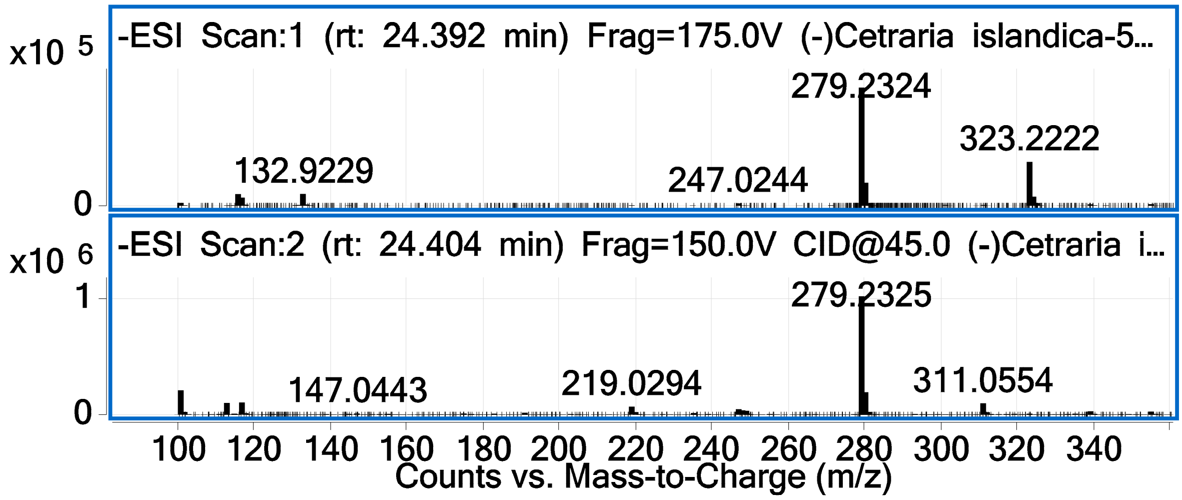

Peak-32

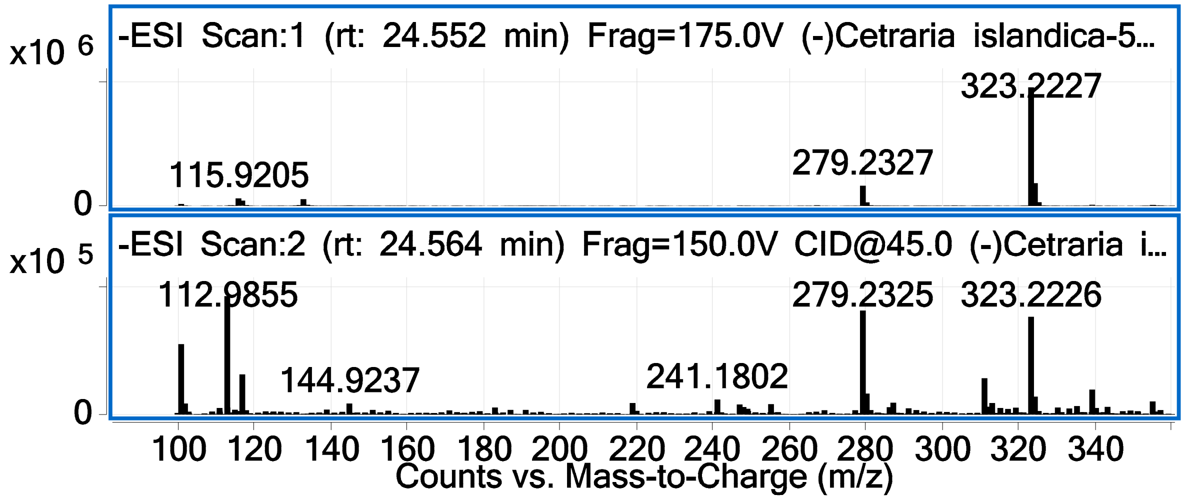

Peak-33

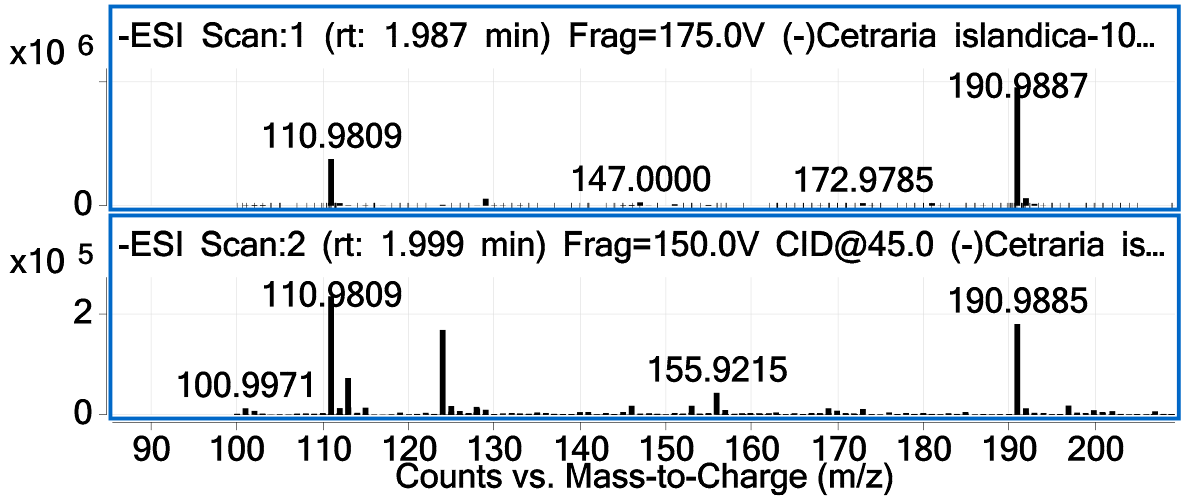

Peak-34

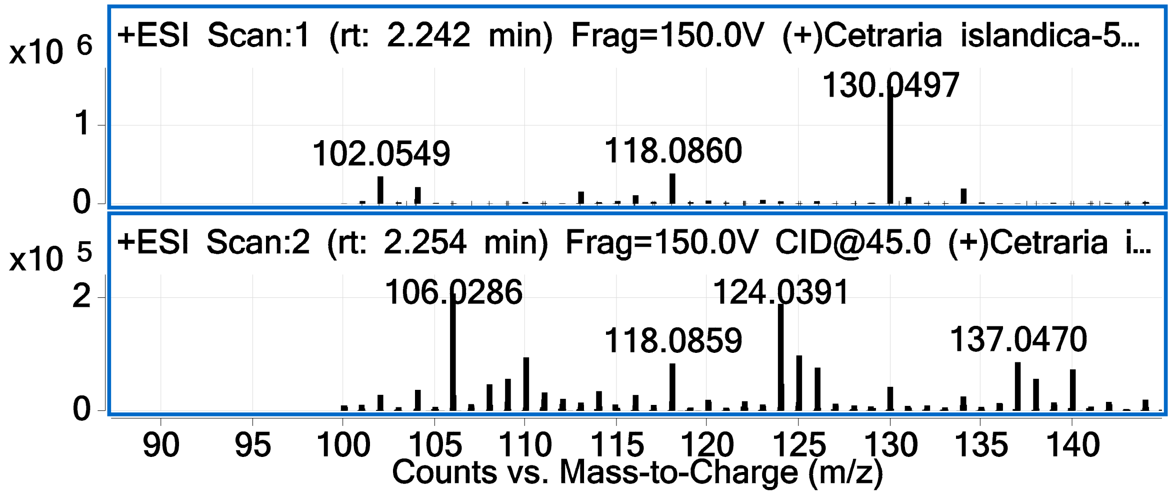

<u>Peak-35</u>

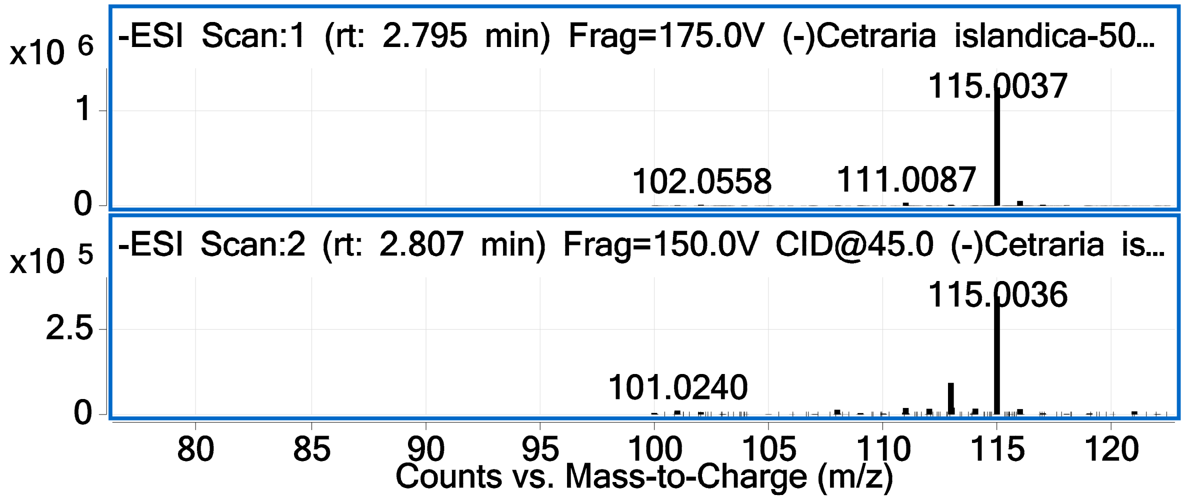

<u>Peak-36</u>

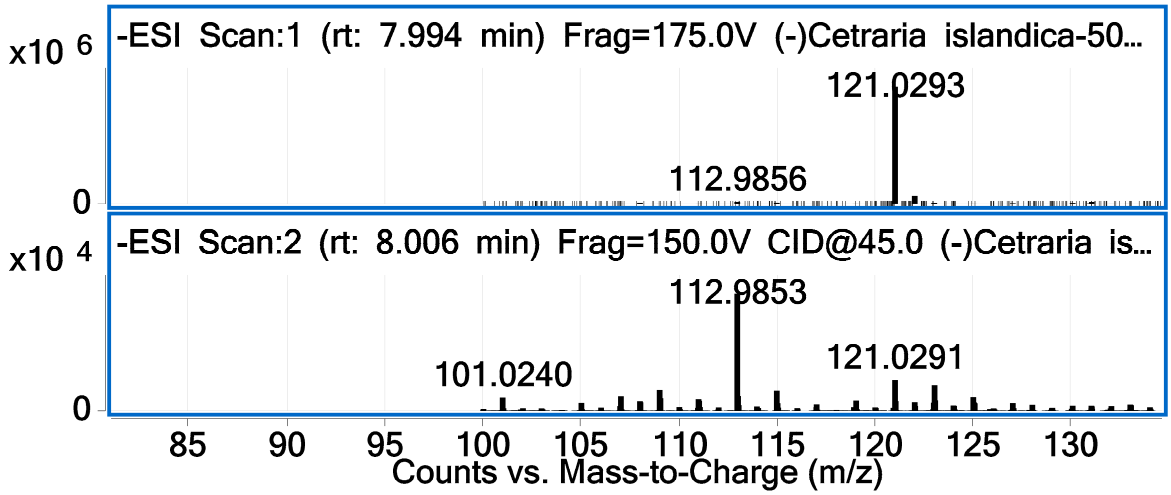

<u>Peak-37</u>

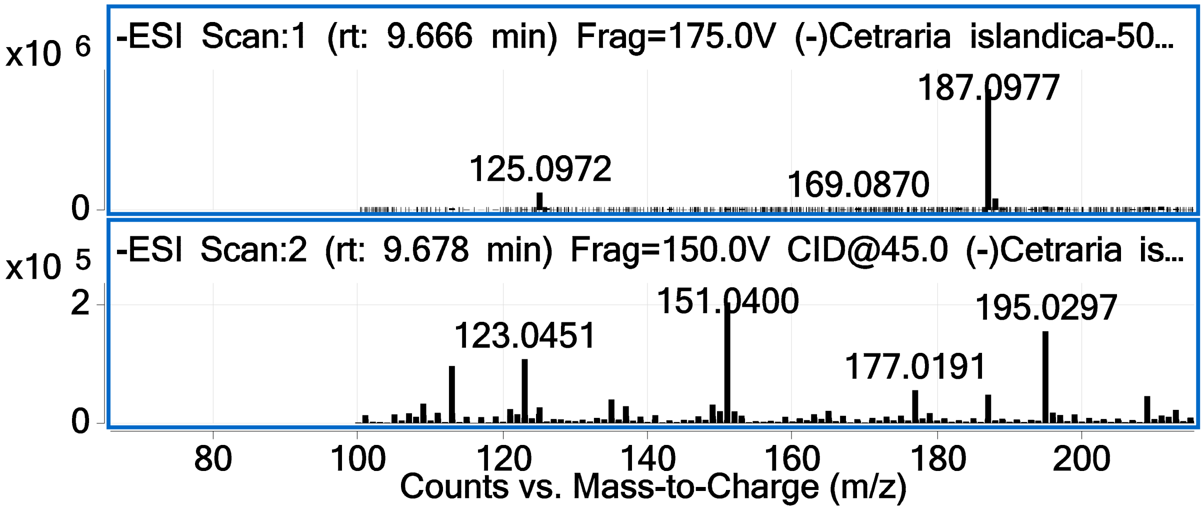

Figure S29. HR-MS/MS spectra of the identified compounds **1-37**
